# High-Order Information Analysis of Epileptogenesis in the Pilocarpine Rat Model of Temporal Lobe Epilepsy

**DOI:** 10.1523/ENEURO.0403-24.2025

**Published:** 2025-05-21

**Authors:** Morteza Mirjebreili, Josu Martinez de Aguirre Ibarreta, Daniele Marinazzo, Laetitia Chauvière

**Affiliations:** ^1^Institute for Cognitive Science Studies, Tehran 1658344575, Iran; ^2^Faculty of Engineering, Mondragon Unibertsitatea, Arrasate Mondragón 20500, Spain; ^3^Department of Data Analysis, Ghent University, Ghent 9000, Belgium; ^4^Institute for Molecular and Behavioral Neuroscience, University of Cologne, University Hospital Cologne, Cologne 50937, Germany; ^5^INS - Institut de Neurosciences des Systèmes, UMR INSERM 1106, Aix-Marseille Université, Marseille 13385, France

**Keywords:** animal study, electrophysiology in vivo, epileptogenesis, higher-order interactions, prediction, temporal lobe epilepsy

## Abstract

Temporal lobe epilepsy (TLE) is a devastating disease, often pharmacoresistant and with a high prevalence of 1% worldwide. There are a few disease-modifying therapies; thus, prevention has become a health priority. The overarching goal of this research project is to highlight the system's dynamics at different stages before TLE onset to identify an early shift in network dynamics trajectory toward disease onset. Researchers often investigate collective brain activity by tracking dynamical interactions of the signal recorded at multiple sites. However, these interactions are usually only computed between pairs of brain regions, at the risk of missing simultaneous interactions of three or more areas, an aspect that is crucial in a networked disease such as TLE. We thus propose to track, on a rich dataset of electrophysiological brain signals recorded within the temporal lobe (TL) of adult male Wistar Han rats, the formation and dissolution of high-order informational multiplets in time during distinct natural behaviors in an animal model of TLE. We identified the informational content of the multiplets as synergistic or redundant. Results identified an early transition of synergistic and redundant multiplets ahead of TLE onset with the predominant involvement of four TL brain regions in generating theta (4–12 Hz) activity. This shift has been shown predominantly during exploration, a theta-dependent behavior, less during rest and sleep. This specific change suggests a shift in communication from an integrated to a segregated network toward TLE onset.

## Significance Statement

Temporal lobe epilepsy (TLE) is a prevalent network disorder that is often pharmacoresistant; therefore, TLE prevention is critical. This research article identifies early signs of TLE by studying collective dynamics within the temporal lobe using an information decomposition technique during natural behaviors. This computational method, beyond pairwise interactions, may thus need to be used to identify early biomarkers for TLE onset.

## Introduction

Temporal lobe epilepsy (TLE) is a prevalent and devastating brain disease that affects ∼1% of the population worldwide (Asadi-Pooya et al., 2017). It is characterized by a latent seizure-free period followed by a seizure-prone period (chronic stage; French et al., 1993; Engel, 1996; Wieser, 2004; Banerjee et al., 2009). TLE is often pharmacoresistant, so preventing TLE appears to be a suitable pathway to embrace rather than treating the disease (The Lancet null, 2019). Toward prevention, we need to identify early signs of the development of TLE onset, a process called epileptogenesis (Dudek et al., 2002; Dudek and Shao, 2003; Dudek and Staley, 2007, 2012; Dudek, 2009; Löscher and Brandt, 2010; Pitkänen and Engel, 2014; Pitkänen et al., 2015). Preliminary results have shown, using coherence in the theta (4–12 Hz) frequency range between pairwise interactions among temporal lobe (TL) brain regions recorded in control and experimental adult rats, that there was a significant decrease in the strength of the coupling from the control stage to late epileptogenic stages, namely, toward TLE onset. The TL network, working as a whole during a theta-dependent behavior, may suddenly shift to a local mode of functioning with fewer long-range interactions, a significant decrease in the strength of coupling in pairwise interactions, and a strengthening of the remaining local interactions. To confirm those results, we focused on collective dynamics beyond pairwise interactions between distributed and highly interconnected TL brain regions using the O-information (Rosas et al., 2019).

Brain statistical interdependencies beyond pairwise metrics consist of functional interactions between three (triplets) or more (quadruplets, etc.) brain regions called high-order interdependencies or multiplets. Studying those higher-order interactions between more than two variables is necessary as a signature of emergent behavior. Information theory provides a convenient framework to achieve this. In this work, we used an information decomposition technique to find multiplets of variables sharing common information and to assign labels of redundancy or synergy to these multiplets. We thus applied this technique to study the network dynamics within the TL, its trajectory, and informational content (redundancy vs synergy) during control versus epileptogenic stages of the animals, during distinct natural behaviors.

The main question of interest that we were investigating with this work was whether the evolution of synergistic versus redundant multiplets could inform on the state of the TL network as an early sign toward the development of TLE onset. Based on a recent study that discovered that synergistic information could constitute a warning sign of a transition toward a more organized state (Stramaglia et al., 2021)—and considering that TLE could be seen as a more organized state than the healthy one—we hypothesized that synergy could be informative in whether the network trajectory is heading toward TLE onset, namely, that it could constitute an early marker of the development of TLE. According to our preliminary results, our working hypothesis was that, ahead of TLE onset, the network would shift from a more integrated network—necessary to effectively process information toward a given outcome or behavioral demand—to a more segregated one. As a more integrated network characterized by high synergy, we expected the transition to a more segregated network to be described by a loss of synergy, which may be compensated by higher redundancy. This shift may be due to the early and persistent deficit in brain network dynamics that we have previously characterized in the theta frequency range during the early stages of epileptogenesis and which correlated with spatial memory deficits (Chauvière et al., 2009). To test our hypothesis, we analyzed electrophysiological extracellular brain signals recorded chronically in awake behaving adult rats during two stages: control versus epileptogenic pilocarpine-treated TLE animals. The pilocarpine model of TLE, well admitted in the field and characterized by a seizure-free period preceding a seizure-prone period, was the TLE model of choice (Curia et al., 2008, Lévesque et al., 2016). We have strong expertise with this model and published pioneer results characterizing in vivo abnormalities before TLE onset (Chauvière et al., 2009). This study is longitudinal, with each rat being recorded in the long run and most recorded rats being their own control (before model induction).

## Materials and Methods

### Animals

Six male adult Wistar Han rats (200–250 g, Charles River Laboratories) were used to perform the electrophysiological recordings analyzed in this study. All tests were performed during the light phase of the cycle, standardized in terms of experimental conditions, and recorded in the first part of the morning. All experimental protocols were approved by the French Institute of Health and Medical Research.

### Electrodes

Tungsten microelectrodes with a 50 μm tip have been used to perform chronic in vivo extracellular recordings in several distributed and interconnected brain regions within the TL. These electrodes were independently moveable using platinum 3D-printed custom–made microdrives (Lab of Physics, Luminy Campus, Marseille, France).

### Experimental design

#### Electrode implantation

Rats were deeply anesthetized with a mixture of xylazine (0.5 mg/kg)/ketamine (1 mg/kg) and placed in a stereotaxic apparatus. Four craniotomies were performed to reach several TL brain regions ranging from the dorsal (AP, −4.8 mm from the bregma; LM, −2 mm [dentate gyrus (DG)], −2.7 mm (CA1), or −3.3 mm (CA3) from the bregma; DV, 4 mm (DG), 2.8 mm (CA1), 3.8 mm (CA3)) and the ventral hippocampus [vHPC; AP, −5.8 mm; LM, −4.5 (CA3) to −5.5 (CA1) mm; DV, −7.8 mm], the median septum (MS; AP, 0.2 mm; LM, −0.2 mm; DV, 4.3 mm), the entorhinal cortex (EC; AP, −5.8 mm; LM, 5.8 mm; DV, −9.0), and the supramammillary nucleus (SuM; AP, −4.8 mm; LM, −1 mm; DV, −8.8 mm), which are distributed and interconnected TL brain regions mainly involved in the generation of the theta rhythm (Chauvière, 2019). Four stainless-steel cortical electrodes were screwed on the skull (two screws in the right and left frontal cortex, one reference, and one ground in the right and left cerebellum). Additionally, six stainless-steel screws were placed on both sides of the lateral part of the skull for the dental cement to hold as it covered up the electrodes. During the surgery, the microdrives were implanted, and the microelectrodes were kept right below the dura matter of the brain. After the surgery, several turns per day were performed to lower the electrodes to the desired brain region and let the brain stabilize a little after each series of turns.

#### Electrophysiological recordings

A Neuralynx recording setup (hardware) was used to perform electrophysiological recordings. The sampling frequency of the setup was 32,556 Hz, and we used up to 16 channels. An event marker option was used to mark in real time on the recording file the animal's behaviors while recording its brain activity. Brain recordings were performed during the animals’ natural behaviors 1 h before and 1 h after the cognitive task in an empty open-field arena (80 cm in diameter, 80 cm high), with a yellow cue card, affixed on the wall. Lighting was directed to the ceiling to provide a homogeneous, dim illumination of the open field. Before the cognitive task (an object recognition paradigm that quantifies both spatial and nonspatial memory), the rat was put in the open field for 1 h to allow it to explore the novel environment without any object. Exploration is crucial for spatial learning and is considered a necessary stage for gathering information about the surrounding environment and for building up a spatial representation of this new environment (Thinus-Blanc, 1996; Thinus-Blanc et al., 1996). After completing the task, we recorded further animal behaviors during 1 h in the same open-field arena without any object, especially sleep. In this manuscript, we did not explore behaviors during the cognitive task due to the high frequency of movement artifacts. In the analyses, epochs of behavior before and after the cognitive task, notably epochs of sniffing and rest, were lumped together. Sleep epochs only occurred after the completion of the task. As specified above, experimental conditions were standardized as much as possible regarding the time of experiments and conditions of experiments. Experiments occurred only in the morning.

#### Longitudinal study

This study is longitudinal by nature. Most of the animals are their own control, with all rats being recorded over several time points [e.g., when the rat was not fit for an experiment after model induction (compare next section), a time point has not been considered in the analysis]. The time points considered in this study are the following: before injections (D-7), which corresponds to the control stage of the animals, namely, before model induction (compare next section), 4 d (D4), 7 d (D7), and 10 d (D10) after model induction, namely, before TLE onset, as well as 14 d (D14) and 25 d (D25) after model induction, namely, during the epileptic (chronic) stage considered in the analysis for comparison.

#### Pilocarpine model

All animals have been subjected to model induction after electrode implantation and electrophysiological recordings at the control stage. Rats received an intraperitoneal injection of pilocarpine hydrochloride (310 mg/kg)—a chemoconvulsivant and muscarinic cholinergic receptor agonist—30 min after an intraperitoneal injection of scopolamine methylnitrate (1 mg/kg; Curia et al., 2008). Status epilepticus (SE) was stopped by diazepam (8 mg/kg) after 40 min. All drugs were obtained from Sigma-Aldrich.

#### Histological procedures

Histological procedures were used to verify the microelectrodes’ placement for each rat. The description of these procedures can be found in Chauvière et al. (2009). For the rat whose tissue was not exploitable for histology (*n* = 1), we statistically compared its results with the results from rats from which we got the precise location of the electrodes. If the results were similar, we have assumed that the electrodes have reached the expected brain region—especially so as the surgeries were performed later, therefore with more experience and consistency in reaching the expected brain regions (adjustments based on the earlier surgeries and related histology)—but if results were diverging, we have discarded this animal from our analyses. No animal has been discarded based on this motive. Histological results can be found in Table 1.

**Table 1.** Histological results of electrode placement

Rat number	01	02	03	04	05	06
Brain regions						
MS	✔	✔	ubt	✔	✔	✔
MS	✔	✔	ubt	✔	✔	✔
Thal	✔	✔	ubt	✔	✔	✔
SuM	✔	✔	ubt	✔	✔	✔
dHPC	✔	✔	ubt	✔	✔	✔
dHPC	✔	✔	ubt	✔	✔	✔
dHPC	✔	✔	ubt	✔	✔	✔
dHPC	✔	✔	ubt	✔	✔	✔
vHPC	✔	✔	ubt	✔	✔	✔
EC	✔	✔	ubt	✔	✔	✔
EC	✔	✔	ubt	✔	NA	✔

This table presents the histological results (cresyl violet histology) of the electrode placement. “ubt,” unusable brain tissue; therefore histological procedures were unfortunately impossible to carry out; compare Materials and methods. NA, not applicable (lack of recording channel).

#### Data collection and analysis

Extracellular signals recorded from our tungsten microelectrodes were amplified (1,000 times), acquired continuously at 32,556 Hz (64-channel DigitalLynx, Neuralynx) at 16 bit resolution and bandpass filtered (0.1 Hz–2 kHz). First, EEG signals have been scrutinized to contain no major artifacts and to be characterized by a good signal-to-noise ratio, then chunked into epochs of behavior which have been marked during the experiment (event markers) and grouped into “sniffing,” “rest” (i.e., awake immobility), or “sleep.” As a final check, each epoch of data was scrutinized to rule out any compromised epoch in terms of noise or artifacts that could have been missed. Next, raw data have been preprocessed using a custom-written MATLAB script. Briefly, the brain signals were reformatted from Neuralynx (.ncs) to MATLAB (.mat) files, reshaped by concatenating the rows to create a 2D matrix of data for each epoch of behavior (time series × channels), downsampled from 32,556 Hz (sampling rate) to 2,000 Hz, and merged into categories of brain regions [namely, regions of interest (ROIs)] when needed. For example, if four channels had been recorded in the dorsal hippocampus (dHPC), these four channels have been averaged and merged into the ROI named dHPC. This grouping was performed to keep the same number of ROIs per chunk (behavioral epoch) of data if the number of valid channels differed (keeping the same example, if two or three valid channels instead of four were found in a few epochs, this had no impact on the maintenance of the ROI named dHPC). This was critical for the computation of the information decomposition method we chose (i.e., the O-information; compare below) where the number of interactions to be considered is crucial and thus needs to remain always the same across epochs of behavioral data as well as across rats and time points. The custom-made script written in MATLAB for preprocessing of the data can be made available upon request. Each 2D matrix consists of several columns, and each column corresponds to one ROI.

#### Subsets of data

We divided our results into two categories of ROIs: Category A and Category B. The reason is that we did not have the same number of ROIs recorded simultaneously at all time points and for all rats, as the method we use implies that the same number of ROIs should be kept over time for all rats to compare the number of high-order interactions (HOIs) among the same ROIs along epileptogenesis. A way to proceed was to analyze distinct categories of rats, with enough rats in each category and enough ROIs. We came up with two categories, containing the same six rats in each category and the following brain regions recorded: Category A includes the thalamus (Thal), the MS, the dHPC, and the vHPC. Category B includes the MS, the SuM, the dHPC, and the EC. Both categories differed in their number of epochs (one epoch exactly) as one epoch of sniffing has been removed from the analysis due to a bad signal-to-noise ratio in one brain region (the EC) in Category B.

#### Behavioral states

Natural behaviors considered in the analysis were the following: sniffing, generating a prominent theta (4–12 Hz) rhythmic activity in awake behaving rats, awake immobility (rest), and sleep. Regarding the latter behavior, only slow-wave sleep epochs have been considered in the analyses since they were more prominent than REM sleep epochs. It is worth noting here that REM sleep is a theta-dependent behavior, similar to sniffing during wakefulness; however, due to the lack of a significant number of epochs, we could not consider REM sleep as a separate brain state in the analysis. Sleep behavior, therefore, includes only slow-wave sleep.

#### Time course of epileptogenesis

To validate the stages and trajectory of epileptogenesis, we looked at continuous raw data and investigated the occurrence of Type A and Type B interictal EEG activity (IA; spike-and-waves) bursts, as described in one of our previous works (Chauvière et al., 2012). Based on the same features, we characterized the occurrence of IA and the occurrence of ictal EEG events, namely, seizure activity. We remind the reader that all preprocessed epochs considered in the present analyses were free of seizure-like events. You can find this analysis in Table 2. Briefly, four rats (among the six) considered in the current study presented a similar time course of epileptogenesis, with mainly Type A IA bursts occurring over D4, D7, and D10. IA bursts were prominent in the dHPC and the EC and sometimes synchronized over all TL brain regions with an inversion of polarity within the dHPC. The remaining two rats, which developed their first spontaneous seizure (SZ1) rather early during epileptogenesis—namely, around D7, therefore earlier than average (usually SZ1 occurred around D10)—had continuous raw data mainly characterized by Type B IA bursts. This is a sign of a network trajectory already set toward TLE onset.

**Table 2.** Visual analysis of IA and ictal-like events (seizure activity) for each rat considered in the present study

Rat number	01	02	03	04	05	06
IA bursts type	Ictal events (seizures, at D7 and at D10); Type B bursts (D7, D10)	Type A IA (D7 and D10)	Type A IA (D7); seizures at D10.	Type A bursts (D7 and D10); Type 2 IA (Type B bursts, D10); ictal events (from D7)	Type A bursts (D4, D7, and D10)	Type A bursts (D4, D7 and D10)
Brain regions	Seizure starting in the EC and propagating in all TL brain regions recorded (*); Type 2 IA in the hippocampus and EC	Synchronized in all TL brain regions, inversion of polarity within dorsal hippocampal layers; EC; dHPC.	EC; generalized seizures.	Seizures usually start in the EC and propagating in all TL brain regions recorded^a^. Type 1 IA either synchronized EC–dHPC or independently occurring in the EC and/or in the dHPC (Type A bursts); Type 2 IA in the dHPC	Synchronized in all TL brain regions, inversion of polarity within dorsal hippocampal layers; EC	dHPC and vHPC

This analysis determines the type of IA bursts—based on the same features as the ones described in Chauvière et al. (2012)—and the seizure activity occurring along epileptogenic stages (namely, D4, D7, and D10), as well as which brain regions they involve. We note that Type B bursts occurred more prominently in two rats which have already undergone early seizure activity. The other rats have a similar pattern of (mainly Type A) IA bursts which characterizes a similar network trajectory along epileptogenesis.

^a^Plus frontal cortex in case of generalized motor seizures.

#### O-information

To assess the amount of joint information in groups of variables, we used the O-information (Rosas et al., 2019). This quantity is defined as the difference between two terms: the total correlation, defined as the difference between the sum of the individual entropies and the joint entropy, and the dual total correlation, defined as the difference between the joint entropy and the sum of all the conditioned entropies. A positive O-information indicates that the system can better be explained by shared randomness (i.e., redundancy; Luppi et al., 2020, 2024; Stramaglia et al., 2021), while a negative one indicates that the system can better be explained by collective constraints (i.e., synergy; Luppi et al., 2024). Entropy can be computed using different estimators. Here, we choose a closed-form expression for the entropy as a function of the determinant of the covariance matrix, with bias correction (Ince et al., 2017). We computed the O-information for all the possible combinations of 3, 4, and 5 signals for each epoch consisting of N signals recorded over T time points. The O-information of each of these multiplets was validated by evaluating the bias-corrected and accelerated confidence intervals of the O-information values via nonparametric bootstrap with replacement (Efron and Tibshirani, 1994; DiCiccio and Efron, 1996; Davison and Hinkley, 1997), checking whether they contained zero. To account for multiple testing, a false discovery rate with a significant value of 0.05 was applied to the *p* values obtained by the proportion of the bootstrap values higher or lower than zero. Likewise, to make sure that each multiplet was significantly redundant or synergistic in its entirety and not just as an effect of containing lower-order multiplets, the confidence intervals of multiplets of order K were confronted with the confidence intervals of the multiplets of order K-1 contained in it and considered significant only in case of nonoverlap. The procedure output was then statistically validated O-information values for multiplets of order 3, 4, and 5, together with the variables composing these multiplets, and the confidence intervals. The code to perform these calculations can be found at https://github.com/danielemarinazzo/HOI.

#### Time domain

The O-information has been computed in the time domain rather than the frequency domain. This decision was motivated by the fact that we did not want to restrict the analysis to one particular frequency band but rather investigate whether our hypothesis whereby there is a shift in the informational content of multiplets at the early stages of TLE was true and prominent during a specific type of behavior, e.g., a theta-dependent behavior. We therefore wanted to study whether our hypothesis could mainly apply to brain regions involved in the generation of the theta rhythm, such as the MS, the hippocampus, the EC, and the SuM, without restricting the analysis to the theta band.

### Normalization of the results

**Figure 1.**
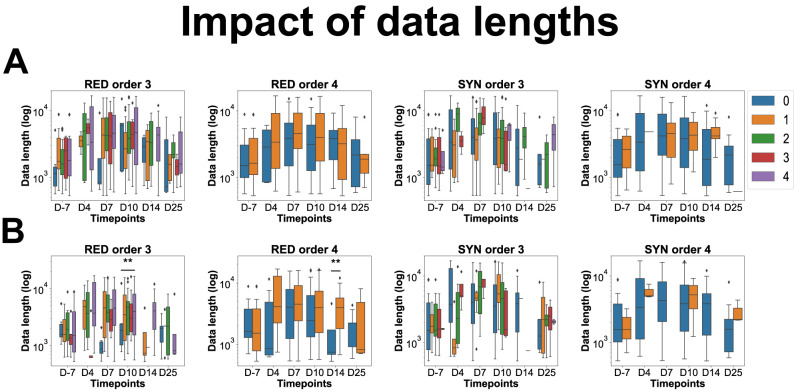
Impact of the data lengths of epochs on the mean number of interactions found per epoch of behavior. Examples of RED/SYN multiplets during sniffing (Extended Data Fig. 1-1 for multiplets during rest and sleep). This figure represents in the *X* axis the time points from D-7 (control stage, before injections) to D4, D7, D10, D14, and D25 post injections; in *Y* axis, the logarithmic of the lengths of epochs considered in our analysis to quantify the mean number of redundant and synergistic third-order interactions (triplets) at each time point, during sniffing in Category A (***A***) and Category B (***B***). This figure shows that, overall, the data lengths of the epochs considered in the analysis have no impact on the number of significant interactions found in those epochs. **p < 0.01 (only cases where data length significantly affects the number of HOIs found in the considered epochs). Note that for fourth-order HOIs (i.e., quadruplets), there is only zero or one HOI, as each category has four brain regions, thus the possibility of several third-order HOIs (triplets) but only one quadruplet. Nb. HOIs, number of HOIs.

The temporal variation in data length across behavioral epochs influences the identification of significant interactions within datasets. As the data length increases, the probability of detecting significant interactions within a given epoch is enhanced, which could introduce an obvious bias in our results. To overcome this bias, we decided to apply normalization procedures. To achieve this, various quintiles were systematically examined to establish optimal boundaries for removing outliers (Wei et al., 2019). The iterative testing of quintile-based outlier removal was conducted until the data length and the number of identified significant interactions reached a point of statistical insignificance. This outcome underscores that variations in data length do not exert a statistically significant influence on the observed quantity of interactions. Upon thorough experimentation, the investigation concluded by selecting the 10 and 80% quintiles as the lower and upper boundaries for each epoch, respectively. This strategic choice not only revealed the insubstantial impact of data length on the number of identified significant interactions but also ensured the preservation of a substantial proportion of the dataset. Adopting these specific quintiles demonstrates a nuanced approach to outlier removal, underscoring the robustness of the methodology in maintaining data integrity while mitigating the influence of temporal variations in data length on the observed interactions (Fig. 1; Extended Data Fig. 1-1). Kruskal–Wallis tests were used to assess these findings’ statistical significance, followed by Conover post hoc tests with Holm *p* value adjustment. This comprehensive statistical analysis aimed to determine whether data length significantly differed across epochs with varying numbers of interactions (0, 1, 2, 3, or 4). The investigation sought to establish whether such variations in data length had a consequential effect on the number of significant interactions detected. This methodological integration underscores the meticulous consideration given to both statistical rigor and data preservation in exploring interactions within the dataset. Another type of normalization has been applied to the data. As the number of behavioral epochs was neither the same at each time point nor for each behavior or rat, the results on the number of interactions have always been normalized by the number of epochs on which the number of interactions has been calculated.

10.1523/ENEURO.0403-24.2025.f1-1 Figure 1-1 Impact of the data lengths of epochs on the mean number of interactions found per epoch of behavior for remaining multiplets and behaviors. This figure is an extension of Figure 1 for the remaining multiplets and behaviors in both categories. This figure thus represents in the X axis the time points from D-7 (control stage, before injections) to D4, D7, D10, D14, and D25 post injections, in the Y axis, the logarithmic of the lengths of epochs considered in our analysis to quantify the number of redundant and synergistic multiplets at each time point, during rest and sleep in Category A (A) and Category B (B). As in Figure 1, this figure shows that, overall, the data length of the epochs considered in the analysis has no impact on the number of significant interactions found in those epochs. **: p < 0.01 (only cases where data length significantly affects the number of HOIs found in the considered epochs). Nb HOIs: Number of HOIs. Download Figure 1-1, TIF file.

#### Signal processing analyses

***Number of HOIs over time***. The count of significant redundant and synergistic interactions was calculated in Python for each order of interactions (namely, orders 3 and 4), and the mean and standard deviation (SD; Wilcox and Rousselet, 2023) for all epochs at each time point were calculated. The mean number of interactions has been depicted in count plots for each order where the radius of the circles is proportional to the number of occurrences.

***Planar hypergraphs***. We have used the following generally available code on GitHub (https://github.com/renzocom/hyperplot) to plot the representation of high-order redundant and synergistic multiplets during each natural behavior and category.

***Regional distribution of HOIs***. The count of HOIs per brain region considered all behavioral epochs, showcasing the tally and the mean and SD. The statistical analysis has been conducted independently for each brain region, offering a comprehensive view of the regional distribution of significant multiplets across behaviors and along epileptogenesis.

***Pearson's correlations***. To plot Pearson's correlation, we plotted the number of redundant and synergistic higher-order interactions for each brain region and at each time point for each behavior and category as scatterplots. The regression line indicates the correlation between the variables, providing a visual representation of the relationships across different brain regions, behaviors, and time points.

***Mean O-information scatterplots***. To generate these plots, values of O-information for each interaction were initially assigned to each respective brain region. For instance, if the dHPC–vHPC–MS third-order interaction had a value of O-information of 2.1, this value was assigned to each brain region constituting the interaction. Subsequently, the mean of these values for each brain region was calculated and depicted as line plots. Univariate distribution of the O-information along epileptogenesis has been depicted using a swarmplot of seaborn.

***Mutual information***. Mutual information between each pair of brain regions—pairwise interactions—was calculated using the 2D matrix of raw signals (time * amplitude) and depicted using heat maps. The resulting values were then averaged and depicted in line plots, offering insights into the average mutual information dynamics across the considered brain regions for each behavior and at each time point. Then, similarly to the univariate scatterplots depicted for the O-information, we established for comparison swarmplots of seaborn depicting mutual information along epileptogenesis based on the averaged values of the mutual information for each brain region and behavior.

***Effect sizes of the changes in O-information and mutual information across time***. To plot the effect sizes of the changes across time for the five different metrics (namely, interaction type: mutual information, redundant triplets, redundant quadruplets, synergistic triplets, and synergistic quadruplets), effect sizes according to the Cohen's *d* formula (compare below, Statistical analyses) were calculated for each behavior and interaction type across time points.

***Colored line or bar plots***. For all figures, the color representation was consistent throughout the plots, with “sleep,” “sniffing,” and “rest” depicted in green, blue, and red, respectively.

#### Statistical analyses

Significant evolution of multiplets over time was analyzed using the nonparametric Kruskal–Wallis test, which was followed by Conover post hoc tests with Holm *p* value adjustment to correct for multiple comparisons if overall significance was found. Pearson's correlation was used to test whether synergistic multiplets correlated with redundant multiplets at any time and for each behavior and category. Cramer's *V* test was used to reveal whether there is a correlation between the shift in redundant and synergistic multiplets during epileptogenesis and the prediction of the SZ1 (correlation for categorical numbers). Effect sizes following Kruskal–Wallis test were calculated using the Eta squared (*η*^2^) formula which is a nonparametric method used to compare three or more independent groups: *η*^2^[*H*] = (*H* − *k* + 1) / (*n* − *k*) where *H* is the Kruskal–Wallis *H* statistic, *k* is the number of groups, and *n* is the total number of observations, namely, the total sample size across all groups. *η*^2^ values range from 0 to 1, with 0 indicating no effect and 1 indicating a strong effect. In practice, the closer the value is to 1, the larger the effect size. Effect sizes following post hoc tests (when the main *p* value was significant) were calculated using the following Cohen's *d* formula: *d* = (M1 − M2) / s_pooled, where M1 and M2 are the means of the two groups being compared and s_pooled is the pooled SD of these groups, calculated as s_pooled = sqrt[((*n*1 − 1)*s*12 + (*n*2 − 1)*s*22) / (*n*1 + *n*2 − 2)], where *s*1 and *s*2 are the SD and *n*1 and *n*2 are the sample sizes of the two groups. In Pearson's correlation, the coefficient *r* is commonly used as an effect size to indicate the strength of the linear relationship between two variables. Lastly, the sample size was determined using the resource equation approach (Unit of Biostatistics and Research Methodology, School of Medical Sciences, Universiti Sains Malaysia, 16150 Kubang Kerian, Kelantan, Malaysia, Wan Mohammad WMZ, Department of Community Medicine, School of Medical Sciences, Universiti Sains Malaysia, 16150 Kubang Kerian, Kelantan, Malaysia, 2017; Karvat et al., 2020): minimum *N* = 10 / (*r* − 1) + 1, with *r* = 6, the number of repeated measurements. Data are expressed as mean ± SD (Wilcox and Rousselet, 2023). The significance level was set at *p* < 0.05. Statistical analysis was performed using Python.

#### Detection of the SZ1

Seizures were detected with semi-continuous EEG recordings since each rat was recorded during the day and overnight. For some rats, video monitoring confirmed generalized motor seizure (Racine Stage 5 seizure). For some rats, TL seizures were detected on the EEG—involving only a few recorded TL brain regions, e.g., starting within the EC and propagating to the dHPC—but did not generalize; those seizures usually occur ahead of the first motor generalized seizure which we characterized as the SZ1. Since we did not only record TL brain regions but also the frontal cortex, EEG activity left no doubt about the occurrence of generalized motor seizures.

#### Code availability

The code for computing the O-information is available at https://github.com/danielemarinazzo/HOI. All the relevant scripts are uploaded at https://github.com/mirjebreili/TLE/tree/main. Explanations are provided for each script in the README file.

## Results

**Figure 2.**
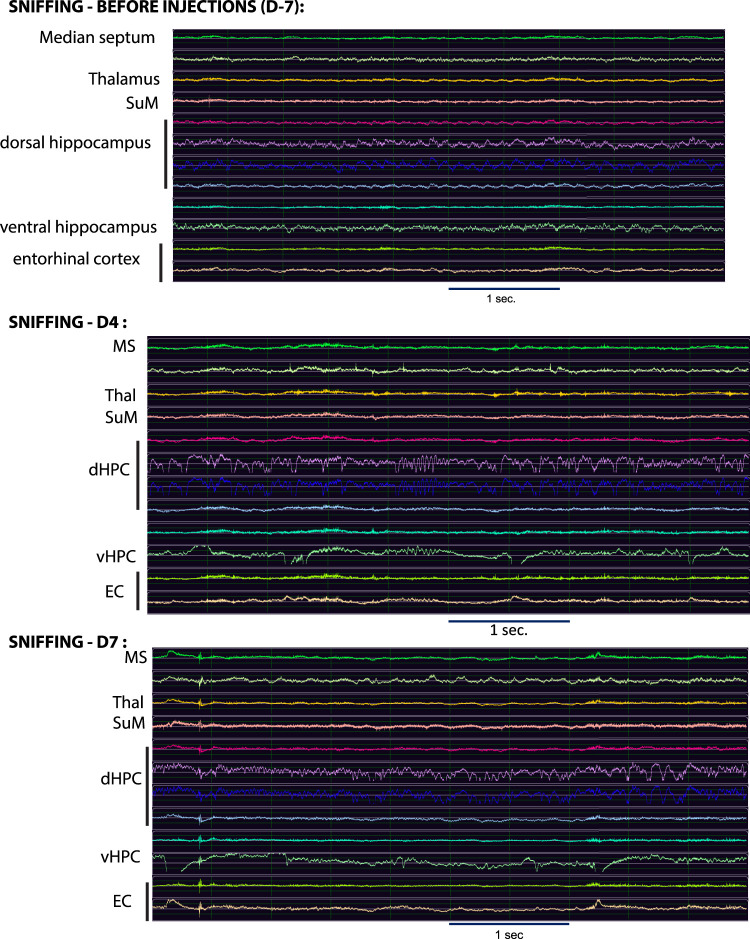
Examples of EEG traces for each brain region considered in the study. This figure depicts examples of EEG traces for each TL brain region considered in the present study before injections (at D-7, i.e., during the control stage), at D4 and D7 during sniffing behavior. *X* axis, time (in seconds); *Y* axis, amplitude (in millivolt). The scale is shown below each set of traces. See Extended Data Figures 2-1–2-5 for examples of EEG traces during sniffing at D10, D14 and D25, as well as during rest and sleep at all time points.

As specified in Materials and Methods section above, the results presented here—namely, the mean number of redundant and synergistic multiplets at each time point and behavior—have been normalized by the total number of epochs considered at each time point and for each behavior. Additionally, we have standardized the length of epochs so that it does not significantly impact the number of significant multiplets found in each epoch considered in the analysis (we refer to Materials and Methods section above for a detailed description of the procedure and to Fig. 1). Examples of EEG traces simultaneously recorded at each time point, for each brain region, and during each behavior are shown in Figure 2 and Extended Data Figures 2-1–2-5.

10.1523/ENEURO.0403-24.2025.f2-1 Figure 2-1 Examples of EEG traces. As an extension of Figure 2, this figure depicts examples of EEG traces for each TL brain region considered in the present study at D10, D14, and D25 during sniffing behavior. X axis: time (in sec); Y axis: amplitude (in mV). The scale is shown below each set of traces. Download Figure 2-1, TIF file.

10.1523/ENEURO.0403-24.2025.f2-2 Figure 2-2 Examples of EEG traces. As an extension of Figure 2, this figure depicts examples of EEG traces for each TL brain region considered in the present study at D-7 (control stage), D4, and D7 during rest behavior (awake immobility). X axis: time (in sec); Y axis: amplitude (in mV). The scale is shown below each set of traces. Download Figure 2-2, TIF file.

**Figure 3.**
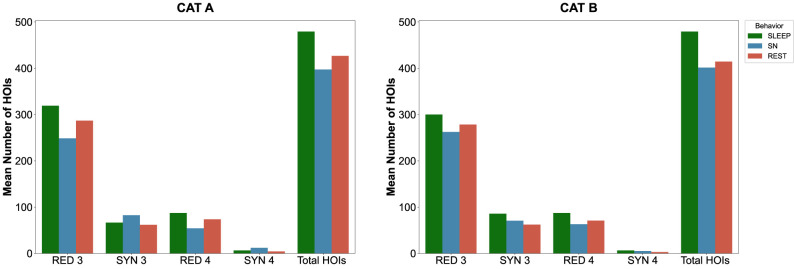
Mean number of HOIs for third- and fourth-order redundant and synergistic multiplets, the total number of epochs considered in the analysis, and the total number of HOIs for each behavior and category during the control stage. These bar plots indicate the mean number of redundant (RED) triplets (RED 3), RED quadruplets (RED 4), synergistic (SYN) triplets (SYN 3), and SYN quadruplets (SYN 4), as well as the total number of HOIs for Category A (CAT ***A***, left) and Category B (CAT ***B***, right) in 100 epochs of sleep (in green), sniffing (SN, in blue), and rest (in red).

10.1523/ENEURO.0403-24.2025.f2-3 Figure 2-3 Examples of EEG traces. As an extension of Figure 2, this figure depicts examples of EEG traces for each TL brain region considered in the present study at D10, D14, and D25 during rest behavior (awake immobility). X axis: time (in sec); Y axis: amplitude (in mV). The scale is shown below each set of traces. Download Figure 2-3, TIF file.

**Figure 4.**
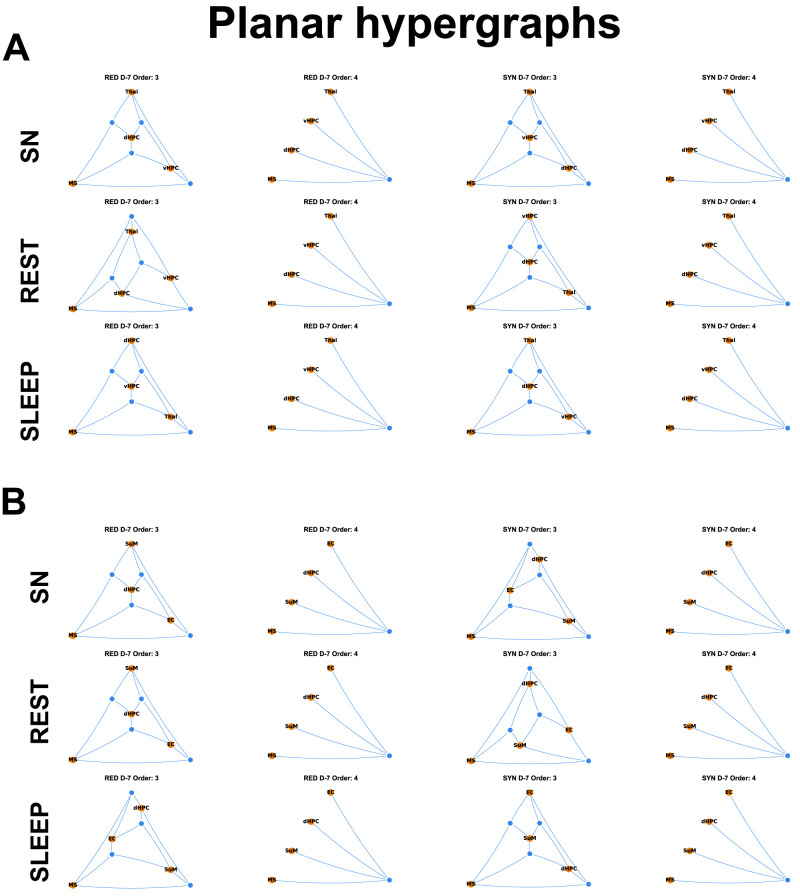
Planar hypergraphs during natural behaviors. Those hypergraphs (cf. https://github.com/renzocom/hyperplot) depict graphical representations of redundant and synergistic triplets (Order 3) and quadruplets (Order 4) during sniffing (SN), rest, and sleep before model induction for Category A (***A***) and Category B (***B***).

10.1523/ENEURO.0403-24.2025.f2-4 Figure 2-4 Examples of EEG traces. As an extension of Figure 2, this figure depicts examples of EEG traces for each TL brain region considered in the present study at D-7 (control stage), D4, and D7 during sleep behavior (slow wave sleep). X axis: time (in sec); Y axis: amplitude (in mV). The scale is shown below each set of traces. Download Figure 2-4, TIF file.

**Figure 5.**
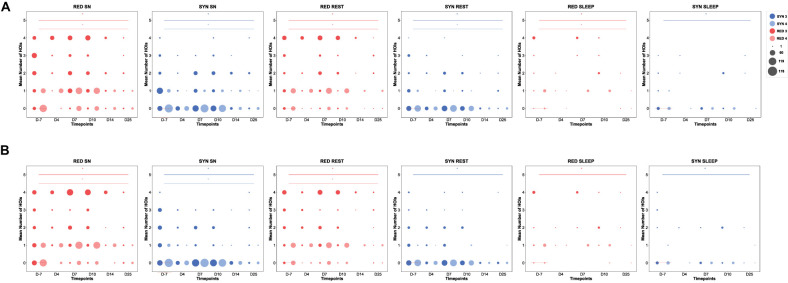
The mean number of redundant and synergistic multiplets during control versus epileptogenic stages for each behavior considered in the analysis. On the *X* axis are depicted the time points from the day before model induction (D-7, control stage) and the following days after model induction, namely, epileptogenic stages for experimental rats, both during the latent period (i.e., ahead of TLE onset, at D4, D7, and D10) and the chronic stage (i.e., epilepsy per se, at D14 and D25). Around D10–D14, on average, the experimental rats all developed their SZ1, marking TLE onset. On the *Y* axis are depicted the mean number of significant HOIs found in all epochs for all experimental rats. Plotted is the mean number of redundant or synergistic high-order TL multiplets along epileptogenesis compared with the control stage for two different orders of interactions (Order 3, triplets of TL brain regions; Order 4, quadruplets of TL brain regions). Those multiplets have been depicted in count plots for each order (3 and 4), where the radius of the circles is proportional to the mean number of interactions in Category A (***A***) and B (***B***). SYN 3, synergistic triplets (dark blue); SYN 4, synergistic quadruplets (light blue); RED 3, redundant triplets (dark red); RED 4, redundant quadruplets (light red). SN, sniffing.

10.1523/ENEURO.0403-24.2025.f2-5 Figure 2-5 Examples of EEG traces. As an extension of Figure 2, this figure depicts examples of EEG traces for each TL brain region considered in the present study at D10 and D25 during sleep behavior (slow wave sleep). Note the absence of epochs at D14 during sleep. X axis: time (in sec); Y axis: amplitude (in mV). The scale is shown below each set of traces. Download Figure 2-5, TIF file.

### Dynamics of HOIs during behavioral states

For Category A, we computed the O-information on a total of 1,082 epochs of behavioral data (645 epochs of sniffing, 374 epochs of rest, and 63 epochs of sleep; *n* = 6 rats; Table 3) over time, including at 7 d before injection (D-7, namely, the control stage). Epochs of data were recorded while the animals *ad libitum* moved and behaved in an empty open-field arena. Likewise, for Category B with 1,083 epochs (646 epochs of sniffing, 374 epochs of awake immobility, and 63 epochs of sleep; *n* = 6 rats; Table 3). The computation of the O-information for each behavioral epoch showed that each natural behavior considered in the analysis was characterized by significant HOIs, i.e., interactions between three (triplets) or sometimes four (quadruplets) brain regions (Table 3). Based on the same number of epochs (ratio calculated over 100 epochs; compare Table 3), overall, more redundant and synergistic multiplets occurred during sleep for Category A and Category B. As each category contained four brain regions, triplets are largely predominant compared with quadruplets (only one possibility per category) for all behaviors and both categories. Those significant multiplets have been classified according to their informational content: redundant or synergistic HOIs. We observed that more redundant multiplets are formed during sleep than during rest or sniffing and more during rest than during sniffing [number (HOI_sleep_) > number (HOI_rest_) > number (HOI_sniffing_)]; this was true for both categories. Regarding synergistic multiplets, more triplets occurred during sniffing than during sleep in Category A and vice versa for Category B. In both categories, synergistic triplets are lower during rest (compare Table 3), with a prominent number of synergistic quadruplets during sniffing, less during sleep, and lowest during rest (Category A; Table 1). In Category B, sleep had the highest number of synergistic triplets while sniffing, and rest had a more similar number of synergistic triplets, with more synergistic triplets during sniffing than rest (Table 3). Synergistic quadruplets followed a similar trend during those behaviors, even though this is less clear since the number of synergistic quadruplets is much lower than the number of triplets. Interestingly, the ratio between redundant triplets versus redundant quadruplets for each behavior and each category is ∼3 or 4. In contrast, the ratio is much higher for synergistic triplets versus synergistic quadruplets, between 6 and 20 (Table 3; Fig. 3). Planar hypergraphs represented these high-order redundant and synergistic interactions during distinct natural behaviors (Fig. 4).

**Table 3.** The total number of epochs and their corresponding number of redundant and synergistic third- or fourth-order multiplets for each behavior and category before injection (at D-7)

Category A					
Sniffing					
Number of epochs	Total number of HOIs (regardless of their informational content)	Number of significant RED HOIs (Order 3: triplets)	Number of significant SYN HOIs (Order 3: triplets)	Number of significant RED HOIs (Order 4: quadruplets)	Number of significant SYN HOIs (Order 4: quadruplets)
645	2,563	1,603	532	350	78
100	397	248	82	54	12
Awake immobility					
374	1,596	1,073	231	276	16
100	426	286	61	73	4
Sleep					
63	302	201	42	55	4
100	479	319	67	87	6
Category B					
Sniffing					
Number of epochs	Total number of HOIs (regardless of their informational content)	Number of significant RED HOIs (Order 3: triplets)	Number of significant SYN HOIs (Order 3: triplets)	Number of significant RED HOIs (Order 4: quadruplets)	Number of significant SYN HOIs (Order 4: quadruplets)
646	2,593	1,696	457	407	33
100	401	262	70	63	5
Awake immobility					
374	1,550	1,041	233	265	11
100	414	278	62	70	3
Sleep					
63	302	189	54	55	4
100	479	300	85	87	6

This table presents the total number of epochs considered in the analysis for each category and natural behavior—namely, sniffing, rest, and sleep—the total number of HOIs, also called multiplets, be it triplets (third-order multiplets) or quadruplets (fourth-order multiplets) and their informational content, redundant versus synergistic, per behavior and category considered in the analysis, during the control stage.

### Early shift of redundant and synergistic multiplets during epileptogenesis

**Figure 6.**
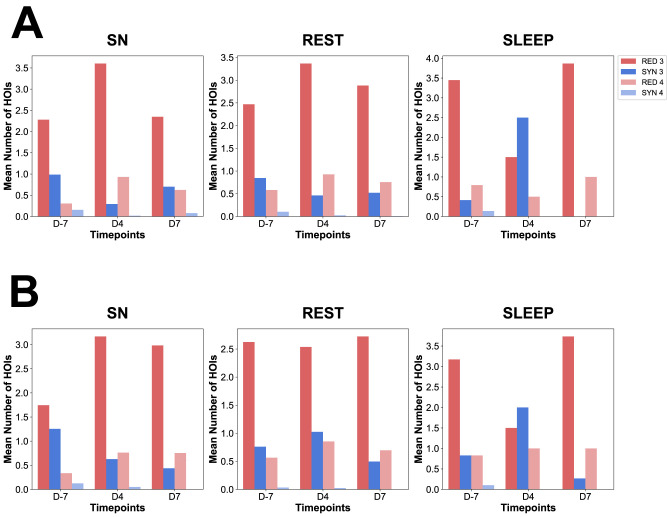
The mean number of HOIs along epileptogenic stages (D4, D7, and D10) compared with the control stage (D-7) for redundant and synergistic multiplets. This figure depicts the mean number of HOIs according to the four possible metrics: RED third order (dark red), RED fourth order (light red), SYN third order (dark blue), SYN fourth order (light blue) during the control stage (D-7) versus epileptogenic stages (D4 and D7) for both Category A (***A***) and B (***B***).

**Figure 7.**
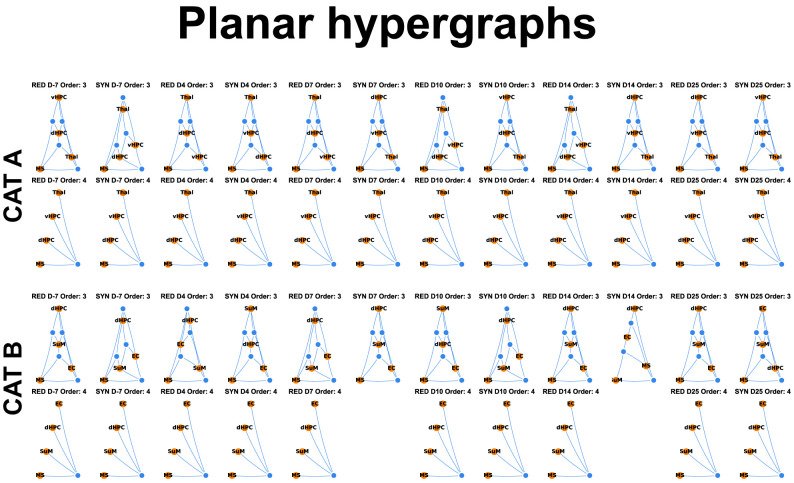
Planar hypergraphs depicting the graphical representation of the evolution of HOIs from the control stage to the epileptogenic stages for sniffing behavior in each category. Extended Data Figure 7-1 for the same graphical representation during rest and sleep.

**Figure 8.**
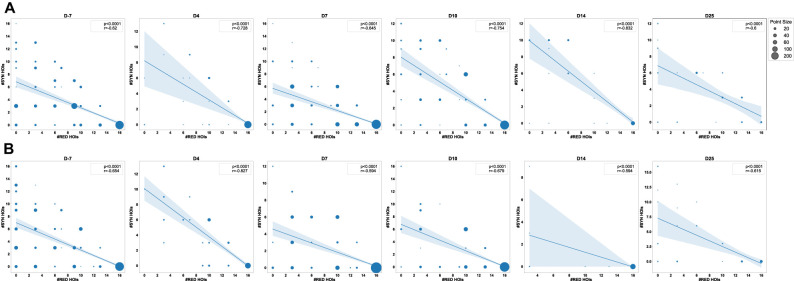
Pearson's correlation plots between the number of redundant and synergistic multiplets during epileptogenesis. This figure depicts the Pearson's correlation between redundant and synergistic HOIs at each time point for all TL brain regions considered in the analysis and all behaviors in Category A (***A***) and Category B (***B***). *X* axis, the number of redundant HOIs; *Y* axis, the number of synergistic HOIs. A regression line indicates the correlation between redundant and synergistic HOIs for each plot, the *p* value the significance of this correlation, and the *r* coefficient the effect size. Nb. HOIs, number of HOIs. See Extended Data Figure 8-1 for the same graphical representation for each TL brain region considered in Category A (***A***) and Category B (***B***).

Next, we investigated the evolution of those high-order redundant and synergistic interactions during natural behaviors along epileptogenesis. Using the Kruskal–Wallis nonparametric test for independent samples revealed that, on average, there is a significant evolution of redundant and synergistic multiplets over time for Category A and Category B (Fig. 5), which allowed us to control for multiple comparisons by performing post hoc tests. Post hoc analyses revealed a significant decrease in the number of synergistic third- (triplets) and fourth- (quadruplets) order interactions at D4 during sniffing, rest, and sleep, accompanied by a significant increase in the number of redundant triplets and quadruplets (compare Table 4 and Figs. 5, 6). Interestingly, this shift tends to normalize by D25 for Category A but persists longer for Category B, particularly for synergistic interactions. The significant changes often peak around D7-D10 before returning to control values by D25, though not all interactions show complete normalization. The shift thus occurred at the early stages of epileptogenesis, as soon as D4 and impairments in the mean number of RED/SYN multiplets often persisted until D14 during sniffing, a theta-dependent behavior, especially for the interactions involving the MS, the dHPC, the SuM, and the EC, namely, an essential quartet in the generation of the theta rhythmic activity (4–12 Hz in rodents). Planar hypergraphs depicting the evolution of HOIs from the control stage to epileptogenic stages are presented in Figure 7 and Extended Data Figure 7-1. Using Pearson's correlation, the number of redundant multiplets has been mainly found to negatively correlate with the number of synergistic multiplets at each time point and during each behavior, for each brain region and for both categories [compare Table 5 for *p* values and correlation coefficients (*r*) as the correlation coefficient itself is the effect size]. This result confirmed that when the number of redundant HOIs increased, the number of synergistic HOIs decreased in our data (Fig. 8; Extended Data Fig. 8-1; compare statistics in Table 6).

10.1523/ENEURO.0403-24.2025.f7-1 Figure 7-1 Planar hypergraphs during rest and sleep depicting the evolution of the mean number of interactions from the control to epileptogenic stages. Those hypergraphs depict the graphical representation of the evolution of HOIs from the control stage to the epileptogenic stages during rest and sleep in Category A (CAT A) and Category B (CAT B), extending Figure 5C, which depicts similar planar hypergraphs during sniffing. Download Figure 7-1, TIF file.

10.1523/ENEURO.0403-24.2025.f8-1 Figure 8-1 Pearson’s correlation plots between the number of redundant and synergistic multiplets during epileptogenesis. This figure depicts Pearson’s correlation between redundant and synergistic HOIs at each time point for each TL brain region and all behaviors in Category A (A) and Category B (B). X axis: number of redundant HOIs, Y axis: number of synergistic HOIs. A regression line indicates the correlation between redundant and synergistic HOIs for each plot, the p-value indicates the significance of this correlation, and the r coefficient (Pearson’s correlation coefficient) indicates the effect size. Nb. HOIs: Number of HOIs. Download Figure 8-1, TIF file.

**Table 4.** The mean number of redundant (RED) and synergistic (SYN) multiplets during sniffing, rest, and sleep across time for Category A and Category B

	Category A											
	Sniffing				Rest				Sleep			
	RED	SYN	RED	SYN	RED	SYN	RED	SYN	RED	SYN	RED	SYN
	Third order	Third order	Fourth order	Fourth order	Third order	Third order	Fourth order	Fourth order	Third order	Third order	Fourth order	Fourth order
D-7	2.280	0.985	0.305	0.155	2.470	0.846	0.581	0.103	3.448	0.414	0.793	0.138
D4	3.603	0.293	0.931	0.017	3.366	0.463	0.927	0.024	1.5	2.5	0.5	0
D7	2.348	0.702	0.624	0.079	2.882	0.521	0.756	0.008	3.867	0	1	0
	Category B											
	Sniffing				Rest				Sleep			
	RED	SYN	RED	SYN	RED	SYN	RED	SYN	RED	SYN	RED	SYN
	Third order	Third order	Fourth order	Fourth order	Third order	Third order	Fourth order	Fourth order	Third order	Third order	Fourth order	Fourth order
D-7	1.745	1.255	0.335	0.125	2.624	0.761	0.564	0.034	3.172	0.828	0.828	0.103
D4	3.169	0.627	0.763	0.051	2.537	1.024	0.854	0.024	1.5	2	1	0
D7	2.983	0.438	0.753	0	2.723	0.496	0.697	0	3.733	0.267	1	0

This table presents the mean number of HOIs from the control stage (D-7) to epileptogenic stages (D4 and D7), highlighting a shift at D4 during sniffing, rest, and sleep, which is more prominent during sniffing in both categories. This number has been normalized by the number of epochs considered at each time point to allow for comparison.

**Table 5.** Pearson's correlation results between the number of redundant multiplets and the number of synergistic multiplets during epileptogenesis

Category A									
Behavior	Time point	MS		SuM		dHPC		EC	
		p	r	p	r	p	r	p	r
Sleep	D-7	0	−0.949	0	−0.965	0	−0.95	0	−0.958
	D4	NA	NA	1	−1	1	−1	1	−1
	D7	NA	NA	NA	NA	NA	NA	NA	NA
	D10	0.003	−0.707	0	−0.936	0	−0.936	0	−1
	D25	1	−1	1	−1	NA	NA	NA	NA
Sniffing	D-7	0	−0.574	0	−0.583	0	−0.513	0	−0.539
	D4	0	−0.679	0	−0.758	0	−0.718	0	−0.652
	D7	0	−0.628	0	−0.626	0	−0.625	0	−0.629
	D10	0	−0.744	0	−0.72	0	−0.74	0	−0.795
	D14	0	−0.742	0	−0.829	0	−0.727	0	−0.82
	D25	0.004	−0.598	0.001	−0.65	0.005	−0.587	0.001	−0.681
Rest	D-7	0	−0.667	0	−0.636	0	−0.534	0	−0.683
	D4	0	−0.699	0	−0.698	0	−0.706	0	−0.722
	D7	0	−0.628	0	−0.651	0	−0.503	0	−0.64
	D10	0	−0.821	0	−0.69	0	−0.68	0	−0.671
	D14	0	−1	0	−1	0	−1	0	−1
	D25	0.389	−0.25	0.105	−0.452	0.011	−0.655	0.461	−0.215
Category B									
Behavior	Time point	MS		SuM		dHPC		EC	
		p	r	p	r	p	r	p	r
Sleep	D-7	0	−0.888	0	−0.849	0	−0.856	0	−0.856
	D4	NA	NA	NA	NA	NA	NA	NA	NA
	D7	0	−1	0	−1	0	−1	0	−1
	D10	0.006	−0.675	0.215	−0.34	0.025	−0.574	0.035	−0.548
	D25	1	−1	1	−1	1	−1	1	−1
Sniffing	D7	0	−0.611	0	−0.601	0	−0.636	0	−0.625
	D4	0	−0.882	0	−0.806	0	−0.853	0	−0.787
	D7	0	−0.573	0	−0.423	0	−0.624	0	−0.6
	D10	0	−0.645	0	−0.533	0	−0.596	0	−0.555
	D14	0	−0.625	0	−0.639	0.006	−0.45	0	−0.6
	D25	0.003	−0.635	0.003	−0.635	0.009	−0.567	0.054	−0.436
Rest	D-7	0	−0.643	0	−0.628	0	−0.607	0	−0.511
	D4	0	−0.856	0	−0.739	0	−0.741	0	−0.78
	D7	0	−0.553	0	−0.636	0	−0.561	0	−0.623
	D10	0	−0.888	0	−0.819	0	−0.836	0	−0.841
	D14	NA	NA	NA	NA	NA	NA	NA	NA
	D25	0.055	−0.544	0.045	−0.563	0.055	−0.544	0.068	−0.52

This table shows the results from Pearson's correlation between the number of redundant multiplets and the number of synergistic multiplets at each time point for each behavior, category, and each TL brain region considered in our analysis. *p* stands for *p* value, and *r* stands for correlation coefficient (also stands for the effect size). NA, not applicable. NA values stand for undefined *r* values, which typically occur when one or both variables (namely, either #RED or #SYN) exhibit zero variance. Specifically, Pearson's correlation coefficient is undefined when at least one of the variables has a constant value, resulting in zero SD. The formula to calculate *r* involves dividing by the product of SD of #RED (SD1) and of #SYN (SD2); thus when the SD of one of the variables is zero, the denominator of the formula becomes zero, leading to an undefined or NaN result (NA value).

**Table 6.** Summary of *p* values and effect sizes (ES) for redundant and synergistic multiplets

Category	Behavior	Type	Order	Time point	*p* value	ES
A	Sniffing	Redundant	Triplets	D4	<0.001	1.119
A	Sniffing	Redundant	Triplets	D7	1	0.053
A	Sniffing	Redundant	Triplets	D10	0.057	0.264
A	Sniffing	Redundant	Triplets	D14	1	0.041
A	Sniffing	Redundant	Triplets	D25	0.487	0.454
A	Sniffing	Redundant	Quadruplets	D4	<0.001	1.475
A	Sniffing	Redundant	Quadruplets	D7	<0.001	0.673
A	Sniffing	Redundant	Quadruplets	D10	<0.001	0.76
A	Sniffing	Redundant	Quadruplets	D14	0.38	0.298
A	Sniffing	Redundant	Quadruplets	D25	1	0.097
A	Sniffing	Synergistic	Triplets	D4	<0.001	0.773
A	Sniffing	Synergistic	Triplets	D10	0.083	0.162
A	Sniffing	Synergistic	Triplets	D14	1	0.074
A	Sniffing	Synergistic	Triplets	D25	0.715	0.478
A	Sniffing	Synergistic	Quadruplets	D4	0.040	0.423
A	Sniffing	Synergistic	Quadruplets	D7	0.186	0.237
A	Sniffing	Synergistic	Quadruplets	D10	1	0.108
A	Sniffing	Synergistic	Quadruplets	D14	0.005	0.537
A	Sniffing	Synergistic	Quadruplets	D25	1	0.298
A	Rest	Redundant	Triplets	D4	0.002	0.619
A	Rest	Redundant	Quadruplets	D4	<0.001	0.772
A	Sleep	Redundant	Triplets	D4	0.020	1.757
A	Sleep	Synergistic	Triplets	D4	0.008	2.542
B	Sniffing	Redundant	Triplets	D4	<0.001	0.966
B	Sniffing	Redundant	Triplets	D7	<0.001	0.862
B	Sniffing	Redundant	Triplets	D10	<0.001	0.909
B	Sniffing	Redundant	Triplets	D14	<0.001	1.256
B	Sniffing	Redundant	Triplets	D25	1	0.061
B	Sniffing	Redundant	Quadruplets	D4	<0.001	0.923
B	Sniffing	Redundant	Quadruplets	D7	<0.001	0.919
B	Sniffing	Redundant	Quadruplets	D10	<0.001	1.041
B	Sniffing	Redundant	Quadruplets	D14	<0.001	1.222
B	Sniffing	Redundant	Quadruplets	D25	1	0.032
B	Sniffing	Synergistic	Triplets	D4	<0.001	0.546
B	Sniffing	Synergistic	Triplets	D7	<0.001	0.792
B	Sniffing	Synergistic	Triplets	D10	<0.001	0.818
B	Sniffing	Synergistic	Triplets	D14	<0.001	1.028
B	Sniffing	Synergistic	Triplets	D25	1	0.037
B	Sniffing	Synergistic	Quadruplets	D7	<0.001	0.518
B	Sniffing	Synergistic	Quadruplets	D10	<0.001	0.429
B	Sniffing	Synergistic	Quadruplets	D14	0.017	0.409
B	Sniffing	Synergistic	Quadruplets	D25	1	0.075
B	Rest	Redundant	Triplets	D14	0.035	0.932
B	Rest	Redundant	Quadruplets	D4	0.005	0.621
B	Rest	Redundant	Quadruplets	D10	0.001	0.569
B	Rest	Redundant	Quadruplets	D14	0.039	0.909
B	Sleep	Redundant	Triplets	D10	0.016	0.83

This table presents the statistics corresponding Results, Early shift of redundant and synergistic multiplets during epileptogenesis. To avoid disrupting the flow of reading, we did not want to clutter the main text with statistics. Instead we decided to include them in a separate table as they included both *p* values and effect sizes.

### Regional distribution of redundant and synergistic multiplets during epileptogenesis

**Figure 9.**
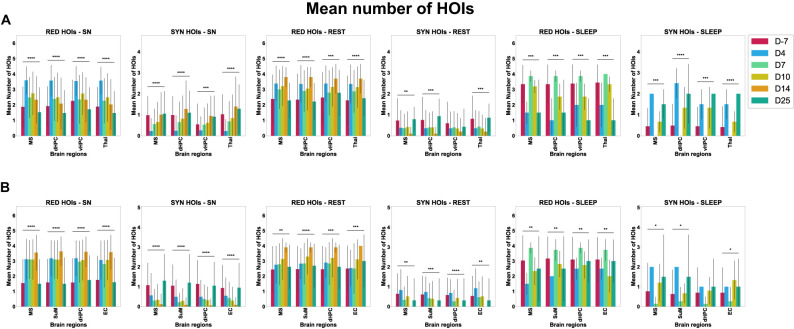
Regional distribution of redundant and synergistic multiplets during control versus epileptogenic stages. These bar plots show the mean number and SD of HOIs at each time point for each brain region, behavior, and category: **A**, Category A; ***B***, Category B. *X* axis, TL brain regions; *Y* axis, the mean number of HOIs.

Investigating the regional distribution of third- and fourth-order redundant and synergistic multiplets over time during each natural behavior (Fig. 9), we showed that the dHPC, MS, SuM, EC, and Thal are most involved in the early shift characterized by a significant increase of redundant interactions and a significant decrease of synergistic interactions. This shift was prominent at D4 during sniffing behavior for the dHPC and the MS in both categories. There was no return to control values at D25, neither for synergy nor redundancy, as effect sizes were negligible despite significant *p* values. Results also showed similar results for the Thal with less consistency, the SuM, and the EC. During rest and sleep, the shift appeared less clear or persistent. Note that there was no epoch of sleep at D14 (compare statistics in Extended Data Fig. 9-1).

10.1523/ENEURO.0403-24.2025.f9-1 Figure 9-1 Statistics corresponding to the third section of the Results entitled “*Regional distribution of redundant and synergistic multiplets during epileptogenesis”*. Download Figure 9-1, DOC file.

### Evolution of triplets and quadruplets during epileptogenesis

**Figure 10.**
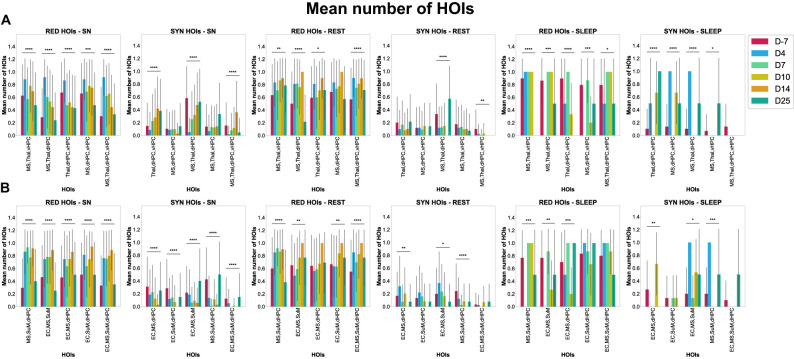
The mean number of redundant and synergistic multiplets during epileptogenic versus control stages in experimental animals for prominent high-order statistical interdependencies. These bar plots depict the mean number and SD of redundant and synergistic multiplets during epileptogenic stages versus the control stage (D-7) in experimental animals for prominent high-order triplets or quadruplets—per behavior and category, Category A (***A***) and Category B (***B***). *X* axis, specific HOIs; *Y* axis, the mean number of HOIs. SN, sniffing.

Next, we looked closely at the TL multiplets that had shown the most significant changes over time. To achieve this, we selected the multiplets significantly impaired in synergy and redundancy across most time points. These multiplets could be triplets or quadruplets. Note that four brain regions constitute each category. Therefore, only one quadruplet can interact per category, compared with triplets. In Category A, five multiplets involving the dHPC, MS, Thal, and vHPC were found to have been significantly impaired along epileptogenesis across all behaviors, both for redundancy and synergy, namely, the following triplets, MS–Thal–dHPC, MS–Thal–vHPC, Thal–dHPC–vHPC, and MS–dHPC–vHPC, as well as the quadruplet, MS–Thal–dHPC–vHPC (Fig. 10; Table 7). During sniffing in Category A, the shift characterized by a significant increase of redundant HOIs and a significant decrease of synergistic multiplets occurred at D4 for most of those prominent interactions, especially for the following triplets, MS–Thal–dHPC, MS–Thal–vHPC, and MS–dHPC–vHPC, and for the MS–Thal–dHPC–vHPC quadruplet. For the triplet Thal–dHPC–vHPC, the shift occurred at D7 regarding redundancy. For those multiplets, their redundant and synergistic informational content got thus impaired mainly at D4 (or D7), persisted until D10, and normalized during the chronic stage at D14 and/or at D25. Similarly, in Category B, five multiplets involving the dHPC, MS, SuM, and EC were found to have been significantly impaired along epileptogenesis across all behaviors, both for redundancy and synergy, namely, the following triplets, EC–MS–SuM, EC–MS–dHPC, EC–SuM–dHPC, and MS–SuM–dHPC, as well as the quadruplet, EC–MS–SuM–dHPC (compare Table 5; Fig. 9). During sniffing, similarly, as in Category A, the shift in Category B mainly occurred at D4 with increased redundant multiplets and decreased synergistic ones, which significantly persisted until D14 (*p* < 0.001). If not at D4, the shift occurred at D7, especially for synergistic quadruplets. We obtained similar results during rest for the following triplet MS–Thal–dHPC and the quadruplet MS–Thal–dHPC–vHPC involving the identical triplet of brain regions in Category A or the triplet MS–SuM–dHPC in Category B; however, this was much less clear if we considered other prominent interactions. During sleep, only the redundant triplet MS–Thal–dHPC in Category A displayed a significant increase at D4 (*p* < 0.001; ES = 2.5), but this effect was not persistent during epileptogenesis. We remind the reader about the absence of epochs at D14 during sleep. The redundant quadruplet was not modified along epileptogenesis, and the remaining prominent triplets mainly displayed a transient increase in redundancy at D10. Regarding synergy, we observed a transient increase at D4 for the following triplets, MS–Thal–dHPC and MS–dHPC–vHPC, and an increase at D10 for the following triplet, Thal–dHPC–vHPC, until D25. In Category B, we obtained similar results (compare statistics in Extended Data Fig. 10-1).

10.1523/ENEURO.0403-24.2025.f10-1 Figure 10-1 Statistics corresponding to the fourth section of the Results entitled “*Evolution of triplets and quadruplets during epileptogenesis”*. Download Figure 10-1, DOC file.

**Table 7.** The mean number of redundant and synergistic triplets and quadruplets during epileptogenic versus control stages in experimental animals for prominent high-order interdependencies

Category A											
Behavior	Time point	MS, Thal, vHPC		MS, Thal, dHPC		Thal, dHPC, vHPC		MS, dHPC, vHPC		MS, Thal, dHPC, vHPC	
		RED	SYN	RED	SYN	RED	SYN	RED	SYN	RED	SYN
Sleep	D-7	0.897	0.069	0.862	0.103	0.897	0.103	0.793	0.138	0.793	0.138
	D4	1	0	0	1	0.5	0.5	0	1	0.5	0
	D7	1	0	1	0	1	0	0.867	0	1	0
	D10	1	0	1	0	0.333	0.667	0.2	0.667	1	0
	D25	0	0.5	0.5	0.5	0	1	0.5	0.5	0.5	0
Sniffing	D-7	0.626	0.138	0.286	0.581	0.675	0.148	0.66	0.103	0.3	0.153
	D4	0.881	0.068	0.915	0.051	0.864	0.085	0.881	0.085	0.915	0.017
	D7	0.57	0.128	0.603	0.251	0.475	0.223	0.687	0.095	0.62	0.078
	D10	0.779	0.123	0.532	0.318	0.519	0.279	0.779	0.097	0.656	0.117
	D14	0.694	0.139	0.444	0.472	0.444	0.417	0.75	0.028	0.444	0.361
	D25	0.476	0.333	0.238	0.524	0.429	0.381	0.476	0.143	0.333	0.048
Rest	D-7	0.633	0.175	0.5	0.333	0.592	0.2	0.683	0.117	0.567	0.1
	D4	0.833	0.119	0.81	0.119	0.81	0.095	0.833	0.119	0.905	0.024
	D7	0.708	0.133	0.817	0.125	0.6	0.167	0.733	0.092	0.75	0.008
	D10	0.867	0.093	0.76	0.147	0.707	0.08	0.773	0.147	0.813	0.027
	D14	0.9	0.1	1	0	0.9	0.1	1	0	0.9	0
	D25	0.786	0.071	0.214	0.571	0.714	0.214	0.571	0.143	0.714	0
Category B											
Behavior	Time point	MS, SuM, dHPC		EC, MS, SuM		EC, MS, dHPC		EC, SuM, dHPC		EC, MS, SuM, dHPC	
		RED	SYN	RED	SYN	RED	SYN	RED	SYN	RED	SYN
Sleep	D-7	0.767	0.2	0.767	0.2	0.7	0.267	0.833	0.133	0.8	0.793
	D4	0	1	0	1	0.5	0	1	0	1	0.5
	D7	1	0	0.867	0.133	1	0	0.867	0.133	1	1
	D10	1	0	0.267	0.533	0.2	0.667	0.667	0.133	0.867	1
	D25	0.5	0.5	0.5	0.5	1	0	1	0	0.5	0.5
Sniffing	D7	0.296	0.424	0.463	0.217	0.458	0.31	0.502	0.286	0.33	0.3
	D4	0.864	0.136	0.746	0.186	0.746	0.186	0.814	0.119	0.763	0.915
	D7	0.933	0.011	0.781	0.062	0.635	0.225	0.635	0.14	0.753	0.62
	D10	0.771	0.111	0.778	0.092	0.752	0.124	0.745	0.072	0.797	0.656
	D14	0.917	0.028	0.889	0.056	0.861	0.028	0.944	0	0.889	0.444
	D25	0.4	0.5	0.25	0.4	0.5	0.25	0.5	0.15	0.35	0.333
Rest	D-7	0.6	0.242	0.65	0.2	0.642	0.167	0.667	0.133	0.55	0.567
	D4	0.854	0.122	0.488	0.366	0.561	0.317	0.634	0.22	0.854	0.905
	D7	0.916	0.025	0.588	0.235	0.588	0.076	0.63	0.16	0.697	0.75
	D10	0.865	0.081	0.77	0.162	0.676	0.203	0.838	0.081	0.824	0.813
	D14	0.9	0	1	0	1	0	1	0	1	0.9
	D25	0.385	0.077	0.769	0.077	0.692	0.077	0.769	0.077	0.769	0.714

This table shows the mean number of redundant and synergistic triplets and quadruplets at each time point, for each behavior and category (A and B).

### Dynamics of the O-information along epileptogenesis

**Figure 11.**
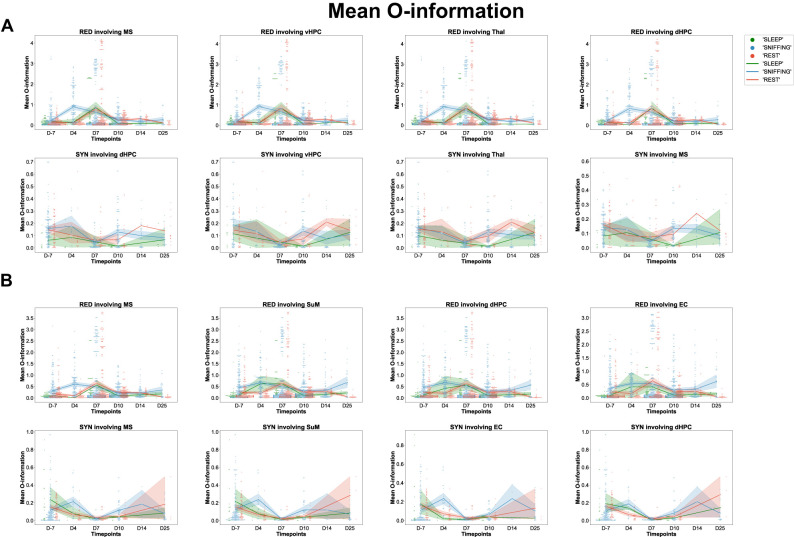
Mean O-information along epileptogenesis compared with the control stage for redundant and synergistic multiplets involving each TL brain region during each behavior and category. Swarmplots of seaborn depicting the evolution of the distribution of the mean O-information values along epileptogenesis (from D4 to D25) compared with the control stage (D-7) during each behavior [sniffing (blue), rest (red), and sleep (green)], for redundant and synergistic multiplets involving a particular brain region according to Category A (***A***) or Category B (***B***).

Next, we investigated the evolution of the O-information along epileptogenesis for each behavior (Fig. 11). In both categories, we observed a significant increase in O-information at D4 for redundant multiplets during sniffing, peaking at D7, and normalizing at D10. Synergistic multiplets revealed a significant decrease in O-information at D4, peaking at D7 and normalizing by D10 (Category B) or D7 until D25 (Category A). During rest, both categories showed a delayed increase in O-information for redundant triplets, peaking at D7 and persisting until D14 or D25. Regarding synergistic triplets across both categories, significant decreases in O-information were observed at D7 with normalization at D10 or D14. No significant changes were found for synergistic quadruplets in either rest or sleep. During sleep, both categories showed a substantial but transient increase in O-information for redundant triplets at D7, returning to control levels by D14 or D25. Let's now look closely at the regional distribution of the changes in O-information across multiplets, as depicted in Figure 10. In Category A, all brain regions showed a significant increase in O-information for redundant triplets during sniffing at D4 and D7 for redundant quadruplets. This increase mainly persisted until D7 or D10 and then returned to normal levels at the chronic stage. In Category B, the O-information of redundant triplets normalized at D7 or D10 and D10 for redundant quadruplets. Regarding synergistic multiplets, all brain regions showed a significant decrease at D7 in both categories; however, no significant change in the O-information for synergistic quadruplets was observed in either category. This significant change was mainly persistent until the chronic stage. This highlights that O-values are highly altered during epileptogenesis, at least during sniffing, especially within the early stages. During rest, redundant triplets in both categories showed a significant increase in O-information at D4 or D7, which mainly persisted until the chronic stage, at D14 or D25; however, we observed no significant changes in O-information for redundant quadruplets ahead of TLE onset.

10.1523/ENEURO.0403-24.2025.f11-1 Figure 11-1 Statistics corresponding to the fifth section of the Results entitled “*Dynamics of O-information along epileptogenesis”*. Download Figure 11-1, DOC file.

Regarding synergistic triplets, all brain regions in Category A showed significant decreases in their O-information at D7. In Category B, the same trend was observed, with all brain areas showing significant changes in O-information during the early stages of epileptogenesis. All brain regions did return to control values of the O-information by D25. During sleep, the O-information of redundant triplets significantly increased at D7 and normalized at D25, except for SuM, which significantly increased at D4, plateaued at D7, and decreased at D10. Regarding redundant quadruplets, there was a strong increase in the O-information at D10 (Category A) or D7 (Category B). Regarding synergy, neither Category A nor B showed significant changes in O-information in triplets or quadruplets during sleep (compare statistics in Extended Data Fig. 11-1).

### Added value of higher-order interactions

One might wonder to what extent higher-order interactions are more informative than pairwise ones. The straightforward answer is that higher-order informational interactions, such as the ones sought in this study, quantify how much extra information is shared between three or more variables. This increase is validated against the informational content of smaller subsets of variables. Now, given a pair of variables, one of which would be the driver and the other the target, one might want to compare the pure dyadic influence with the many-body effects due to the remaining variables in the network. In the information theory framework, this is motivated by a critique of the transfer entropy (James et al., 2016): if one believes that a dyadic network accurately models a complex system, then one implicitly assumes that polyadic relationships are either unimportant or nonexistent. To address this issue, assessing the relative importance of these HOIs compared with dyadic relations, a decomposition of the transfer entropy was recently proposed (Stramaglia et al., 2024). Instead of conditioning the transfer entropy on all processes except the driver and the target, as in its fully conditioned version (*T_f_*), or not conditioning at all, as in the pairwise approach (*T_p_*), the method searches for both the multiplets of variables that maximize information flow (*T_M_*) and those that minimize it (*T_m_*). This provides a decomposition of the transfer entropy into unique (*T_m_*), redundant (*T_p_* − *T_m_*), and synergistic (*T_M_* − *T_p_*) atoms. The algorithm searches all the variables for the first one to be tentatively used as a conditioner. Subsequently, one variable is added at a time to the previously selected ones to construct the set of conditioning processes that either maximize or minimize the transfer entropy. The criterion for terminating the greedy search for conditioning processes, minimizing (maximizing) the transfer entropy, is to stop when the corresponding decrease (increase) of the transfer entropy is compatible with a null distribution obtained by surrogates. In the present study, we focused on the expansion of zero-lag interactions: we then adapted the framework described above to decompose mutual information.

To achieve this, we selected an epoch with four variables. The candidate regions to start with were the MS and the dHPC. Anatomically, the MS sends synaptic input to the dHPC to generate hippocampal theta rhythmic activity (4–12 Hz) locally, which coordinates those brain areas during theta-dependent behavior and cognitive processes (Chauvière, 2019; Takeuchi et al., 2021a,b). The pairwise mutual information was indeed high between these two regions. Performing the decomposition described above, we observed a sharp drop in the pairwise mutual information when adding the other two variables: the vHPC and the Thal. The contribution of higher-order interactions to the pairwise mutual information was higher than 98%, providing a strong motivation for investigating these latter (data not shown).

**Figure 12.**
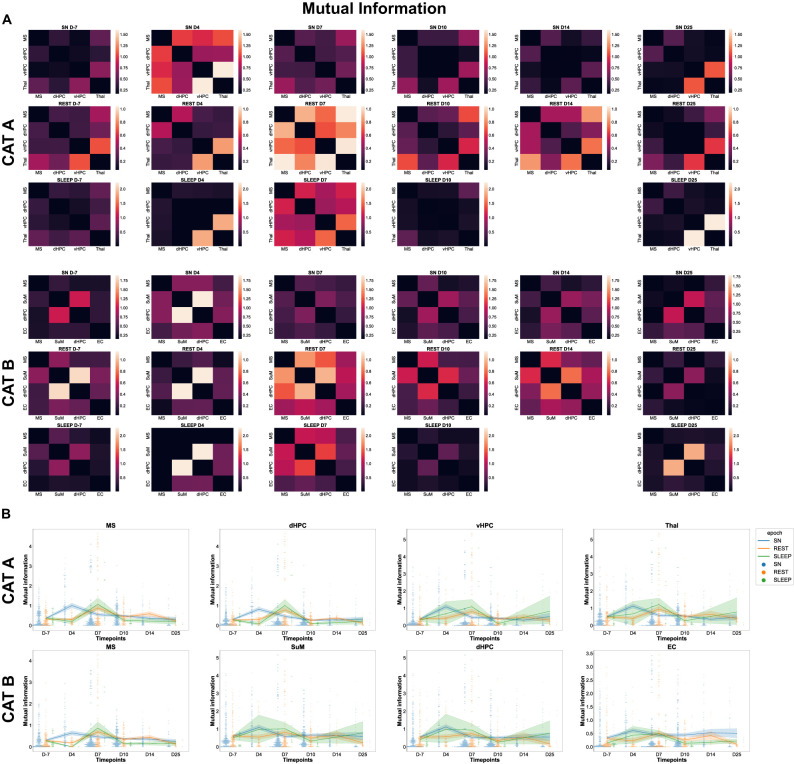
Mutual information between pairwise interactions of TL brain regions at each time point for each behavior and category. ***A***, Heat maps depicting mutual information between all possible pairwise interactions at each time point for each behavior (sniffing, rest, and sleep) and category (A and B). Color bars have the same axis per behavior across time but different limits among behaviors, otherwise creating a loss of information. ***B***, Swarmplots depicting the evolution of the distribution of the mutual information between pairwise interactions (involving a particular brain region) along epileptogenesis compared with the control stage (D-7) for each behavior and category.

In the current study, to investigate whether early alterations along epileptogenesis could be highlighted as well by looking at pairwise interactions, we computed mutual information between pairs of TL brain regions and plotted the related heat maps for each pair of brain regions (Fig. 12*A*); in addition, the related distribution of mutual information along epileptogenesis was plotted as a swarmplot of seaborn, and its average values were plotted as a line plot for each brain region involved in all possible pairwise interactions during each behavior (sniffing, rest, and sleep; Fig. 12*B*). To allow for comparison, we have presented the results obtained from the mutual information analysis in the same way as those obtained from the O-information analysis (Fig. 12*B*). Note that informational content (as redundant or synergistic) cannot be discriminated against for mutual information. Kruskal–Wallis nonparametric statistics revealed a significant increase of mutual information at D4 during sniffing compared with the control stage for both categories. This significant increase at D4 was observed for each brain region involved in pairwise interactions (Fig. 12*B*) and for all pairwise interactions considered in the analysis of Category A and Category B, with a more persistent increase in mutual information for Category B except for MS–SuM which displayed no shift at D4. During rest, the mutual information was increased only during the chronic stage, i.e., after TLE onset, in both categories. More specifically, if we closely looked at the regional distribution of the mutual information impairment, we found that during rest, there was a significant and transient increase at D7 only for pairwise interactions involving the MS in both categories. Surprisingly, the mutual information of the pairwise interactions involving the dHPC and the SuM during rest was significantly altered only later during the chronic stage, namely, at D25. In Category A, only a few pairwise interactions involving the MS displayed higher mutual information at the early stages of epileptogenesis, either at D4–D7 (MS–dHPC and MS–Thal) or later at D10 (MS–vHPC). In Category B, most pairwise interactions involving the MS displayed a significant decrease in mutual information at D4 [MS–SuM; MS–dHPC (but small effect) and MS–EC] or a significant increase at D7 for pairwise interactions involving the dHPC (SuM–dHPC, dHPC–EC, and MS–dHPC). This early shift at D4 was mainly transient, with further sporadic modifications of the mutual information compared with the control stage. During sleep, in both categories, there was a significant increase in the mutual information at D7 compared with the control stage. Looking at pairwise interactions, we observed a significant increase at D7 for pairwise interactions involving the MS and the EC in both categories or later at D10 for pairwise interactions involving the ventral and the dHPC. Specifically, pairwise interactions involving the MS and the dHPC showed significant changes in mutual information, mainly at D7 in both categories (Fig. 12; Fig. 12-1).

10.1523/ENEURO.0403-24.2025.f12-1 Figure 12-1 Statistics corresponding to the mutual information discussed in the sixth section of the Results entitled “*Added value of higher-order interactions”*. Download Figure 12-1, DOC file.

**Figure 13.**
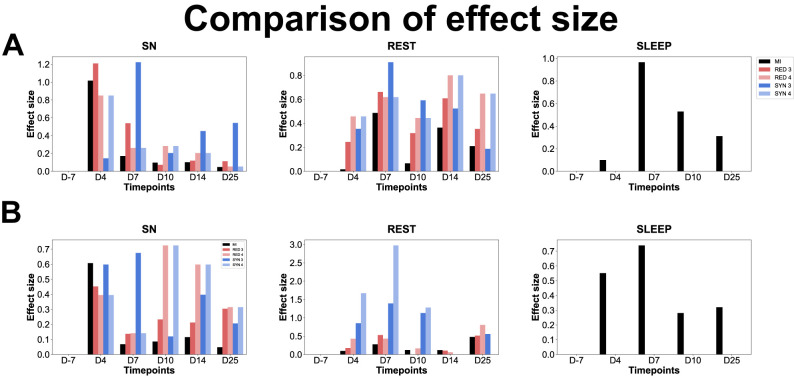
Comparison of the effect sizes of the changes in mutual information and O-information. Interaction information (equal to O-information for *n* = 3) already tells us how much information there is beyond mutual information. We were looking at mutual information to see whether we also observed some modulations over time when using it. We compared the effect sizes of the changes in the two quantities across time. For that, we computed the effect sizes of the changes in the five metrics, namely, mutual information (MI, black) and the four metrics of the O-information [RED third order (dark red), RED fourth order (light red), SYN third order (dark blue), and SYN fourth order (light blue)] along epileptogenesis, for each behavior and category (***A***, Category A; ***B***, Category B). Note the absence of effect size for the SYN metrics (SYN 3 and SYN 4) during sleep in both categories since the Kruskal–Wallis test did not exhibit any significant change in O-information related to the control stage in both categories (thus, no post hoc tests were run).

To further inform the reader about how (much) these measures of O-information versus mutual information are associated with modulations in brain activity, in Figure 13 we reported the effect sizes (from Cohen's *d*) of the statistical test “Measure X changes over time (the different days),” where “Measure X” is pairwise mutual information or synergistic/redundant O-information of Orders 3 and 4. Results showed that changes in redundant and synergistic triplets are very informative at D4 and D7, respectively, during sniffing but also rest, mainly at D7 for multiplets in Category A, thus during the early stages of epileptogenesis. In Category B, we obtained similar results, with a prominent effect size of the changes in synergistic triplets at D4 and D7 during sniffing and, additionally, prominent effect sizes of the changes in redundant and synergistic quadruplets during sniffing at D10 and of synergistic quadruplets during rest at D4 and D7. Mutual information only displayed a prominent effect size of its changes at D4 during sniffing for pairwise interactions in both categories (Extended Data Fig. 12-1).

### Prediction of the SZ1 marking TLE onset

Lastly, we investigated whether the significant shift in the informational content of HOIs from the control stage to the epileptogenic stages occurring mainly at D4 or D7 in each experimental rat can predict the SZ1. We addressed this question by studying whether there is a significant correlation between the shift in higher-order statistical interdependencies and SZ1, e.g., if the significant increase of redundant multiplets and the significant decrease of synergistic multiplets that we observed at D4 could be predictive of an earlier TLE onset marked by the early occurrence of SZ1 than if the shift occurred at D7 or is even not significant (Table 6). To address this question, we used the Cramer's *V* correlation which measured an association between two categorical variables [in this case, “early” shift (at D4) vs “late” shift (at D7, D10, or later); “early” SZ1 vs “late” SZ1, for each experimental rat] using a *χ*^2^ test. This test does reveal a *medium* correlation during sniffing behavior—considering the shift in both redundant and synergistic multiplets—in Category B (involving the dHPC, MS, SuM, and EC; *V* = 0.354); however, no correlation was found in Category A (involving the dHPC, MS, Thal, and vHPC; *V* = 0) except for synergy during rest (Category A). We found no correlation either during rest or sleep (*V* ∼ 0; Table 8).

**Table 8.** Shift in redundant and synergistic multiplets related to the day of the SZ1 of all experimental rats considered in the analysis

Rat number	SZ1	SNIFFING				REST				SLEEP			
		Category A		Category B		Category A		Category B		Category A		Category B	
		RED	SYN	RED	SYN	RED	SYN	RED	SYN	RED	SYN	RED	SYN
01	D8	NS	NS	NS	NS	NS	NS	NS	D10	NS	NS	NS	NS
02	>D10	D14	D7	NS	NS	NS	NS	NS	NS	NS	NS	NS	NS
03	D10	D4	D7	D7	D7	NS	NS	NS	NS	NS	NS	NS	NS
04	D7	D10	D7	D10	D10	NS	NS	NS	NS	D10	D10	NS	NS
05	>D10	D4	D4	D4	D4	D4	D4	D7	NS	NS	NS	D7	D7
06	>D10	NS	NS	NS	NS	NS	NS	NS	D25	NS	NS	NS	NS

Significant shift in redundant (RED) and synergistic (SYN) multiplets during each behavior (sniffing, rest, or sleep), for each category (A or B) and experimental rat related to the day of their SZ1 to investigate whether an early shift in the informational content of higher-order interactions could be predictive of an early occurrence of SZ1, thus of an early TLE onset. NS, not significant. D7 is considered an early SZ1, D10 a standard SZ1, and >D10 a late SZ1.

## Discussion

In this study, we aimed to investigate whether there is a shift in the informational content of higher-order interactions at the early stages of TLE during natural behaviors (sniffing, rest, and sleep) in the TL of chronically implanted adult rats. We computed the O-information across time and behaviors for two categories of four TL brain regions using chronic electrophysiological data. We hypothesized that the network would shift from a more integrated network to a more segregated one ahead of TLE, marking a shift toward disease onset.

### Reversed informational content ahead of TLE onset

In the present study, we identified a significant decrease in synergistic multiplets and a significant increase in redundant multiplets during natural behaviors, especially during sniffing, a prominent theta-dependent behavior, but also during rest and sleep, as soon as the early stages of epileptogenesis, namely, ahead of TLE onset. This early change in the informational content of HOIs occurred as soon as D4 across behaviors. It is as if the network newly rendered epileptogenic has shifted from an integrated to a segregated way of processing information (Tononi et al., 1994). Synergistic information naturally processed by the brain and especially by the TL during a given behavior has been dramatically altered, and the brain may compensate by strengthening its redundant information at the local level, rendering its remaining information available within local sources more robust, hence redundant, at the expense of a more effective way of processing information (Luppi et al., 2024). Indeed, in a previous study, we showed that the theta rhythm was significantly impaired at the early stages of epileptogenesis (D4), and this correlated with a deficit in spatial memory (Chauvière et al., 2009), as we know that theta cycles constitute a window of opportunity for relevant multimodal afferent inputs to be potentiated and irrelevant stimuli to be depotentiated (Huerta and Lisman, 1993) due to a tight schedule between excitatory and inhibitory transmission (Chauvière, 2019). It is, therefore, not surprising that altered theta cycles in terms of power and frequency will have a dramatic impact on the way that information is processed within the TL and, thus, on the informational content of the interactions between several TL brain regions that the theta rhythm usually coordinates. As Luppi and colleagues wrote in their latest review (Luppi et al., 2024), “Redundancy instead provides robustness, as the over-representation ensures that information will remain available if any one source is disrupted,” we can easily understand and speculate that in the case of an epileptogenic network with underlying altered brain network dynamics arising early during the development of TLE, an increase of redundancy is thus expected to guarantee that information—at least at the local level and between the remaining multiplets—“will remain available” if any of the TL brain regions recorded or the brain dynamics used to coordinate them during a given behavior such as sniffing is significantly altered.

### Normalization of the informational content of multiplets at D25

Our current results showed a normalization of redundant and synergistic multiplets at D25, which we could explain by the fact that the network came back to its initial state and tagged the definitive transition from a reversible to an irreversible state toward TLE; in other words, precipitated its impaired trajectory toward TLE. This normalization at D25 during a theta-dependent behavior such as sniffing nevertheless contrasts with the persistent deficit in theta power and frequency previously reported (Chauvière et al., 2009). This discrepancy could be explained by the fact that the impaired TLE network may use novel brain dynamics to coordinate TL brain regions during those newly irreversible epileptic conditions. Those novel brain dynamics may be a network signature that characterizes an established (irremediable/immutable) pathological state, in that case, an epileptic (TLE) one (Le Van Quyen et al., 2003).

### Echoing a dynamic ahead of seizure onset?

Our findings before TLE onset align with past results (Le Van Quyen et al., 2003). The researchers hypothesized that ictogenesis—namely, the process that leads to epileptic seizures in terms of brain dynamics and functional networks—could be explained by the following phenomenon: while the interictal state is characterized by large-scale dynamics of normal brain functioning, which involves multiple brain regions related to the epileptic focus, the subsequent preictal state represents a loss of synchronization between those brain regions and cortical regions as well as between themselves, thus creating a state of local hyperexcitability with the epileptic focus. In other words, the preictal state lost the large-scale brain dynamics, losing large-scale influences of normal brain functioning with the epileptogenic focus, which inhibitory control may maintain. This hypothesis, ahead of the onset of an epileptic seizure, is in line with our hypothesis ahead of the onset of TLE as they both assume a shift from an integrated to a segregated network state, with more excitability at the local level. Our current results align with this hypothesis: an increase in redundant multiplets and a loss of synergistic HOIs. Other research articles using connectivity-based network approaches and intracranial EEG also highlighted such a network transition from the interictal to the ictal state, in particular using a measure of a node's influence within a network of the intracranial EEG correlation graph in frequency space (Burns et al., 2014; Bernabei et al., 2023). Additionally, the analysis of time-varying dynamical connectivity computing the “neural fragility” metric showed that not only was this metric useful to locate the epileptogenic zone in epileptic patients but also, and most interestingly for our study, was it increasing during epileptogenesis in an acute in vivo animal model of epilepsy (Li et al., 2017, 2021; Ehrens et al., 2020). These findings suggest that it may provide similar mechanisms during the transition characterizing the interictal to the ictal state, both during the seizure-free (i.e., ahead of TLE onset) and the seizure-prone TLE period, and confirm a transition from a “balanced” (integrated) to an “unbalanced” (segregated) network, as our current results suggest, with a decrease of synergy and an increase of redundancy during the early stages of epileptogenesis.

### Dynamics of redundant and synergistic HOIs during natural behaviors

In our current work, we noticed a high number of redundant HOIs compared with synergistic ones. According to Luppi et al. (2020, 2024), simple processes are less characterized by synergistic interactions that are more representative of complex cognitive functions (Luppi et al., 2020, 2024). The present study investigated informational multiplets during natural behaviors without cognitive demand, which can explain the high number of redundant (over synergistic) multiplets. More specifically, the computation of the O-information has revealed that there was a higher number of redundant multiplets during sleep (sleep > rest > SN) for both triplets and quadruplets and in both categories and a higher number of synergistic multiplets during sniffing (SN > sleep > rest, Category A) or sleep (sleep > SN > rest, Category B) for both triplets and quadruplets. This discrepancy could be explained by the fact that rest behavior—consisting of awake immobility of the animal—may involve less information processing of combined (complex) information and thus less synergistic information than redundancy, which consists of shared copies of the same information. In contrast, sniffing, a theta-dependent behavior, and sleep may require more complex information processing to process information that has either been encoded during the day and needs to be potentiated or depotentiated (in the case of sleep) or which is currently getting encoded (in the case of sniffing where the animal is probing its environment). This requirement during sleep and sniffing for more information processing may, therefore, demand more synergy than during rest (Luppi et al., 2024) and, in the case of sniffing, less redundancy than during rest (but more redundancy as well during sleep). However, to the best of our knowledge, redundant and synergistic information has not yet been characterized during natural behaviors, only during cognitive processing in nonhuman primates and human subjects (Luppi et al., 2020, 2024); thus our current original article is the first one investigating this question.

### Synergy and redundancy during pathological conditions

Considering the dual total correlation, a generalization of Shannon's mutual information (Shannon, 1948; Rosas et al., 2019; Herzog et al., 2022) has investigated multiplets during two neurodegenerative conditions (Herzog et al., 2022). Researchers found that higher-order functional EEG interactions and fMRI brain signals could reliably characterize and predict these conditions. Hyper- or hypoconnectivity of distinct networks and hypoconnectivity in the delta or gamma band significantly characterized both neurodegenerative conditions. In our current study, which we performed in the time domain, we have not looked yet at the frequency domain. Still, we expect a hypoconnectivity for synergistic multiplets and a hyperconnectivity for redundant multiplets in the theta (4–12 Hz) band. We hypothesize that the MS may be the driver. The targets may be the dHPC and the EC (in case of triplets) as well as the SuM or the Thal, which provide a rhythmic theta frequency input to both the hippocampus and the EC through synaptic transmission and thus likely contribute to theta synchrony between them which is crucial for navigation (Buzsáki, 2005, 2006; Dragoi and Buzsáki, 2006; Foster and Wilson, 2006; Chauvière, 2019; Takeuchi et al., 2021b). Synergy captures the information of the whole (integrated network), namely, the joint state, which researchers completely missed when they only studied bivariate interactions (i.e., pairwise metrics).

In contrast, bivariate interactions do, in general, well represent redundant interactions. In an aging study, Gatica et al. (2021) showed that older subjects, using functional magnetic resonance imaging, were characterized by more redundant high-order brain interdependencies, which the researchers interpreted as less diverse activation in distinct brain regions by comparison with younger participants (Gatica et al., 2021). Researchers identified in those older subjects a “redundancy core,” composed of specific brain regions that may be the source of associated deficits in working memory and executive control (Gatica et al., 2021). In our study, we did not notify any particular “redundancy core” or “synergy core” as both informational contents involved the same brain regions per category and behavior; nevertheless, specific HOIs are prominent, which remain the same for redundancy and synergy—namely, three triplets and a quadruplet per category (compare Fig. 9)—which may constitute specific hubs within the network. These hubs—constituted by distributed and highly interconnected TL brain regions involved in the generation of theta rhythm, which coordinates these regions during cognitive processes and during theta-dependent behavior such as sniffing—may be the source of cognitive deficits. During epileptogenesis, these hubs, coordinated by an altered theta rhythm as early as D4, may give rise to impaired coordination, altered HOIs between those regions, and reversed informational content, thus disrupting information processing at the origin of cognitive deficits during task demands.

### Informational multiplets and IA

IA consists of a pathological spike-and-wave activity that frequently occurs in subjects with epilepsy in between their epileptic seizures but also in experimental models ahead of TLE onset as a sign of an epileptogenic network. In previous work, we identified and quantified a specific dynamic of IA during epileptogenesis in pilocarpine-treated adult rats (Chauvière et al., 2012). This work has defined two types of IA spikes (Type 1 and Type 2) based on morphological features such as spike amplitude, spike duration, wave amplitude, and wave duration, which defined two types of IA bursts (Type A and Type B). Starting with Type 1 IA, which appeared 1 to 2 d after SE in an uninterrupted way, with the duration spent in Type 1 IA activity decreasing from Day 1 to Day 7, Type 2 IA duration increased from Day 4 to Day 14, with, interestingly, a different dynamics which shifts around the SZ1 marking TLE onset but also the same time spent in either Type A or Type B clusters, before D7 and at D40 (Chauvière et al., 2012). Most epochs we analyzed in the present work (except at D-7) contain IA in one of the considered channels (i.e., brain regions). Readers will recall that, apart from two ROIs (the Thal and the SuM), we have grouped the channels that form the other ROIs (e.g., the dHPC and the EC); for example, ROI3 corresponds to the dHPC and usually regroups three or four recording channels. Therefore, we could not discriminate between epochs with or without IA, as there were not enough epochs to be considered in the analysis without IA in any channel to establish significant comparisons based on robust statistics.

Nevertheless, we have conducted a visual analysis to monitor IA activity along epileptogenesis for each rat. This analysis showed that most rats (4/6) followed the standard IA path described above (as in Chauvière et al., 2012). However, two other rats that developed earlier seizure activity displayed earlier Type B bursts, translating into a network whose trajectory precipitates earlier toward TLE onset. Interestingly, the increase in redundant multiplets and the decrease in synergistic multiplets occurred at D4 for the sniffing and rest behaviors and, on average, at D7 for sleep behavioral epochs. If we try to superimpose these two dynamics—redundant/synergistic multiplets versus IA—we could hypothesize that Type 1 IA spikes and Type A IA bursts that arise soon after the initial brain insult (i.e., the SE in our model), utilize resources, mainly inhibitory resources, used to generate local dynamics such as theta brain activity. This conflict of resources may reinforce the network reorganization (remodeling) that arises from the initial brain injury—the latter being at the origin of a lesion of ∼10% neuronal loss in the hippocampus and EC (cf. Chauvière et al., 2009)—and may precipitate even more the TL network trajectory into epileptogenic states. This network reorganization gives rise to altered network dynamics, such as theta impairment and to an abnormal way of processing information, as described in the present study, looking at redundant and synergistic high-order statistical interdependencies, probably at the origin of cognitive deficits. As explained above, information processing occurs primarily through the coordination via theta cycles, with theta also coordinating long-range functional connectivity between remote brain regions, so we can easily imagine that an alteration in those theta cycles would have a critical impact on the way information is processed as well as on high-order TL brain metrics. In general, there was less IA during sleep (cf. Chauvière, 2010), so it does not appear surprising that the network may not be responsive as quickly as for behaviors when IA is more present (such as sniffing or rest) and thus may precipitate earlier an altered network trajectory, processing of information, and synaptic transmission toward epileptogenic states.

### Mutual information versus O-information

Considering higher-order interactions has several motivations. First of all, we have a setting in which the regions are not entirely disjoint units but most likely share anatomical and functional information. Looking at shared information and the nature of this information is then a logical and needed step. Then, pairwise and conditioned approaches, directed (transfer entropy) and undirected (mutual information), can suffer from confounders, leading to false positives and false negatives when shared information exists among the variables. Higher-order informational interactions are intrinsically robust to this issue. Finally, all functional connectivity approaches involve an epistemological bridge between the level of the target properties under investigation (the activity of neural populations and their interactions) and the level of observables and their statistical dependencies (Reid et al., 2019). In this sense, it is only natural to suppose that the functional interactions in the brain are not limited to pairwise ones. Moreover, this cross-order study presents several advantages by looking at the data at the pairwise (through mutual information) level and higher-order interactions (through the O-information; Neri et al., 2024), taking care of correctly identifying these latter as providing novel information above and beyond the pairwise ones.

Globally, if we paralleled the results from the computation of the mutual information and the O-information, the former highlighted a significant shift mainly at D7 or D10. At the same time, the latter revealed a significant shift, mainly at D4 or D7, considering averaged values during each behavior across both categories. The mutual information analysis showed a significant change at D4 if we closely looked at pairwise interactions during sniffing involving specific brain regions, especially the dHPC and the MS. We can conclude from this original work that the computation of the O-information into redundant and synergistic higher-order multiplets complemented the computation of the mutual information between pairwise metrics by identifying an earlier shift toward the development of TLE and by providing a better understanding of the study of network brain dynamics along epileptogenesis, namely, a significant increase of redundant HOIs and a significant decrease of synergistic multiplets which highlighted a transition from an integrated to a segregated network. The computation of higher-order statistical interdependencies between brain network dynamics, and particularly of synergistic multiplets, is therefore critical and, in our opinion, should become a standard analysis in TLE prevention from an initial brain insult.

### Transition mode: less dominated by synergy, more by redundancy

According to a recent study published by Varley and colleagues (Varley et al., 2023), redundancy-dominated or synergy-dominated modes can be seen as distinct “ways” used by the brain to perform information processing according to current behavioral demands. In the present study, we can speculate that the epileptogenic process shifts how the brain processes information while the behavioral demand remains the same. Indeed, during epileptogenesis, for a given behavior, say sniffing, rest, or sleep, the way used by the brain to process information shifts from a mode that is less dominated by synergy and more dominated by redundancy. What may thus happen is that the network, which an initial brain insult (triggered by the SE) starts to reorganize, slowly puts in place epileptogenic processes, and gives rise to impaired brain dynamics such as a disrupted theta rhythmic activity. This network reorganization may generate aberrant information processing at the origin of the emergence of cognitive deficits correlated with the theta rhythm impairment in a previous study (Chauvière et al., 2009).

### Limitations of the study

The present work used a rat model to identify a biomarker of epileptogenesis toward TLE onset prediction. The pilocarpine rat model is well identified and well admitted in the field and characterizes the pathophysiology of human TLE relatively well, but not entirely, as with any model. As the English statistician Georges Box rightly stated, “All models are wrong, but some are useful.” In that sense, the pilocarpine rat model helped us to investigate the seizure-free period that precedes TLE onset, which is a period that is not exploited in TLE patients. However, the model does not fully represent the human TLE pathophysiology. As TLE is difficult to treat, aiming at preventing it is a suitable pathway to embrace. So far, the seizure-free period can only be studied with intracranial EEG in an animal model.

Related to this model, one may wonder whether the informational changes and eventual normalization cannot reflect in part recovery from prior anesthesia and provoked SE in addition to epileptogenesis. First, control rats were given a week to recover from electrode implantation under anesthesia, and before each experiment, electrophysiological signals were checked for the occurrence of expected oscillatory activity; for example, hippocampal theta rhythm during sniffing and REM sleep, as well as hippocampal ripples during awake immobility and slow-wave sleep. Additionally, we were careful not to use anesthesia to, e.g., reconnect the animals with the preamplifier as we realized that urethane/halothane alters theta rhythm for a few minutes after use, and this could constitute a bias if the animals were recorded during this time. We, therefore, never recorded EEG signals until all the expected physiological rhythms were observed as soon as the animals were recorded. Second, induced SE was the trigger mimicking the initial acute brain insult such as a traumatic brain injury, a stroke, or febrile seizures (Klein et al., 2018), which led to mild neuronal loss, epileptogenesis, and TLE onset in our model (Curia et al., 2008; Chauvière et al., 2009). Consequently, the mechanisms put in place by the induced SE may be necessary to generate the initial trigger of epileptogenesis that occurs in most TLE patients and, thus, may participate in the buildup of changes in TL network dynamics.

### Conclusion

Higher-order interactions complement pairwise metrics to study the development toward TLE onset at the early stages; therefore, it is critical to consider interactions beyond pairwise dynamics. Particularly, investigating synergistic multiplets in at-risk subjects may inform on a shift of the network trajectory toward disease onset. Synergy is usually reviewed as being more effective than redundancy, to the point of being sometimes considered the only meaningful quantity. We also found a correlation between the early shift in the informational content of HOIs (including a decrease in synergy) and the development of the SZ1 marking TLE onset during sniffing in Category B. Synergy can thus constitute an early marker of the development of TLE, which may be relevant when studying subjects who had suffered an initial brain insult—e.g., a moderate traumatic brain injury or a stroke—or who had suffered an initial epileptic seizure and who need to be explored with EEG.

#### Perspectives

Further studies will be necessary to characterize with even more specificity the extent to which higher-order synergistic interactions can predict the shift of the network trajectory toward TLE onset. The time course of epileptogenesis will be thoroughly characterized in individual rats; IA and ictal activities will be continuously monitored during epileptogenesis to specifically inform on the network trajectory followed by each rat toward TLE onset. This way, we aim to strengthen the prediction of TLE onset using higher-order interactions beyond the fourth order.

## Synthesis

Reviewing Editor: Catherine Schevon, Columbia University Decisions are customarily a result of the Reviewing Editor and the peer reviewers coming together and discussing their recommendations until a consensus is reached. When revisions are invited, a fact-based synthesis statement explaining their decision and outlining what is needed to prepare a revision will be listed below. The following reviewer(s) agreed to reveal their identity: Steven Tobochnik, Sridevi Sarma. Note: If this manuscript was transferred from JNeurosci and a decision was made to accept the manuscript without peer review, a brief statement to this effect will instead be what is listed below.

The concept of temporal lobe epilepsy networks is commonly invoked but we lack standard definitions for these networks or understanding of complex interactions. This study uses information theory analysis to provide insights into the high-order informational dynamics associated with TLE, highlighting an early diagnostic potential for interventions. This approach is innovative as it extends beyond traditional pairwise analysis, seeking a deeper understanding of how brain regions collaborate or operate independently in response to epilepsy onset. By focusing on multi-regional and multi-order interactions, this work advances our understanding of how network organization precedes epileptic onset, marking a shift from network integration to redundancy as a significant early-stage hallmark of TLE. The results help to understand TLE networks and have significant value for computational modeling of TLE. Some additional clarification would strengthen the study.

1. Do the authors observe effects of multiplets by simply clustering on a connectivity matrix derived from pair-wise correlations? For example, if 3 regions as a triplet have a negative O-info value, do all three regions have high pairwise correlations? Although the reviewers appreciate that the authors assess mutual information, but the utility of O-info in this application is unclear. While it is interesting as a quantity - is it really need it? Alternatively, could the authors examine the connectivity matrix (and clusters within the matrix) over epileptogenesis?

2. Several studies use dynamic network models which capture n-to-n interactions in the time domain. The authors need to consider this literature (below) and discuss how the O-info differ from these approaches. See refs below that discuss these approaches.

Quantitative approaches to guide epilepsy surgery from intracranial EEG

JM Bernabei, A Li, AY Revell, RJ Smith... - Brain, 2023.

Linear time-varying model characterizes invasive EEG signals generated from complex epileptic networks A Li, KM Gunnarsdottir, S Inati, K Zaghloul, J Gale... - 2017 39th Annual International Conference of the IEEE ..., 2017

3. Limitations of the reliance on rat model, which not fully represent human TLE pathophysiology and complex statistical adjustments necessary to control for variable data lengths across behavioral epochs need to be discussed.

4. There are some sentences that are difficult to parse or not grammatically correct. Authors are encouraged to edit for grammar. For example, this is a sentence that is hard to parse:

example in lines 688-690: "Next, we closely looked closely at the TL multiplets which were the most significantly altered, be it triplets or quadruplets (note that each category is constituted by four brain regions, therefore only one quadruplet can possibly interact per category, compared to triplets)."
