# Supplementary figures and images for: High-Order Information Analysis of Epileptogenesis in the Pilocarpine Rat Model of Temporal Lobe Epilepsy
Source: eNeuro. 2025 May 21;12(5):ENEURO.0403-24.2025. doi: 10.1523/ENEURO.0403-24.2025 (PMC12121938; doi:10.1523/ENEURO.0403-24.2025)

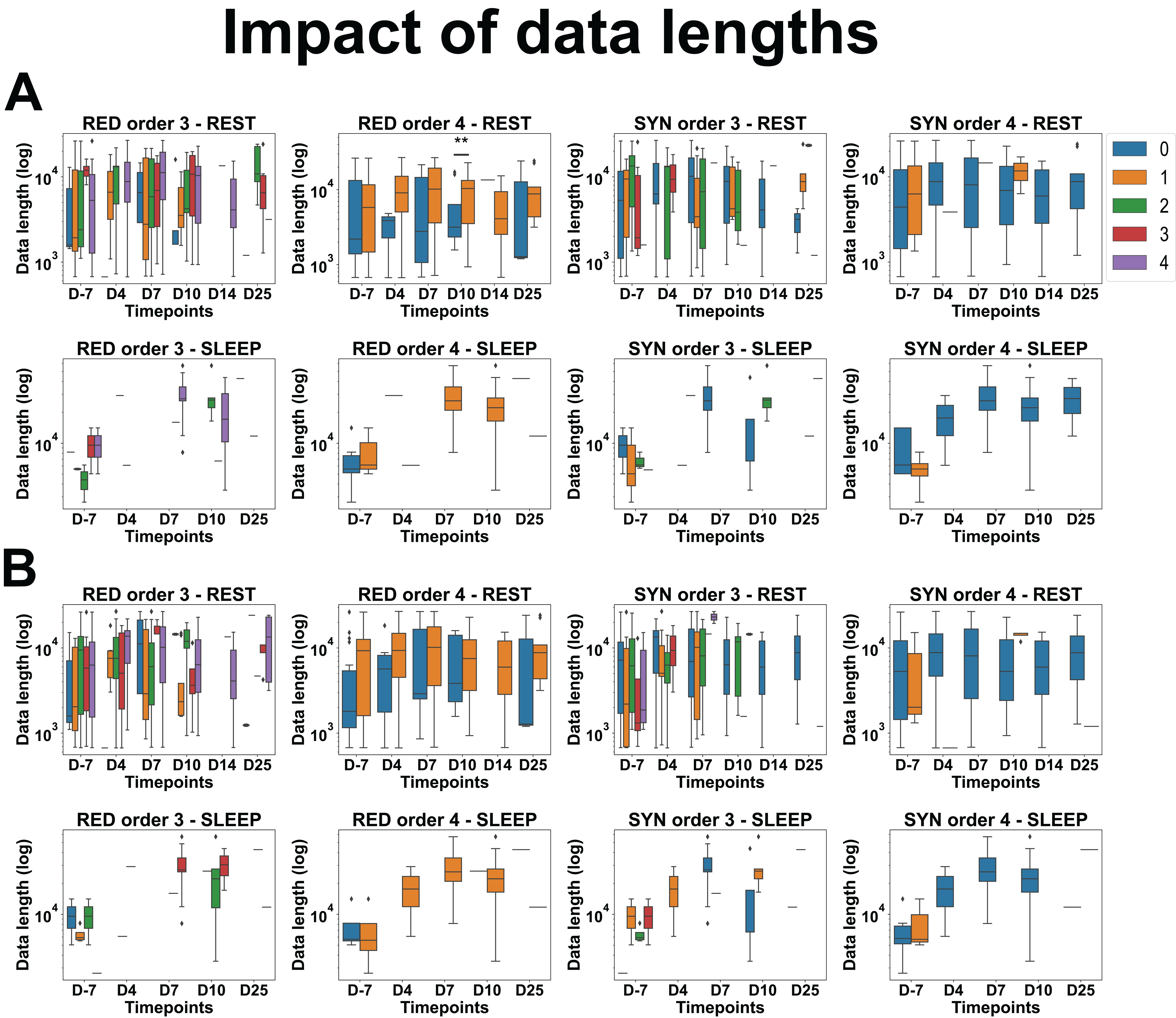

Supplement: Figure 1-1 — Impact of the data lengths of epochs on the mean number of interactions found per epoch of behavior for remaining multiplets and behaviors. This figure is an extension of Figure 1 for the remaining multiplets and behaviors in both categories. This figure thus represents in the X axis the time points from D-7 (control stage, before injections) to D4, D7, D10, D14, and D25 post injections, in the Y axis, the logarithmic of the lengths of epochs considered in our analysis to quantify the number of redundant and synergistic multiplets at each time point, during rest and sleep in Category A (A) and Category B (B). As in Figure 1, this figure shows that, overall, the data length of the epochs considered in the analysis has no impact on the number of significant interactions found in those epochs. **: p < 0.01 (only cases where data length significantly affects the number of HOIs found in the considered epochs). Nb HOIs: Number of HOIs. Download Figure 1-1, TIF file. [file eneuro-12-ENEURO.0403-24.2025-s003.tif]

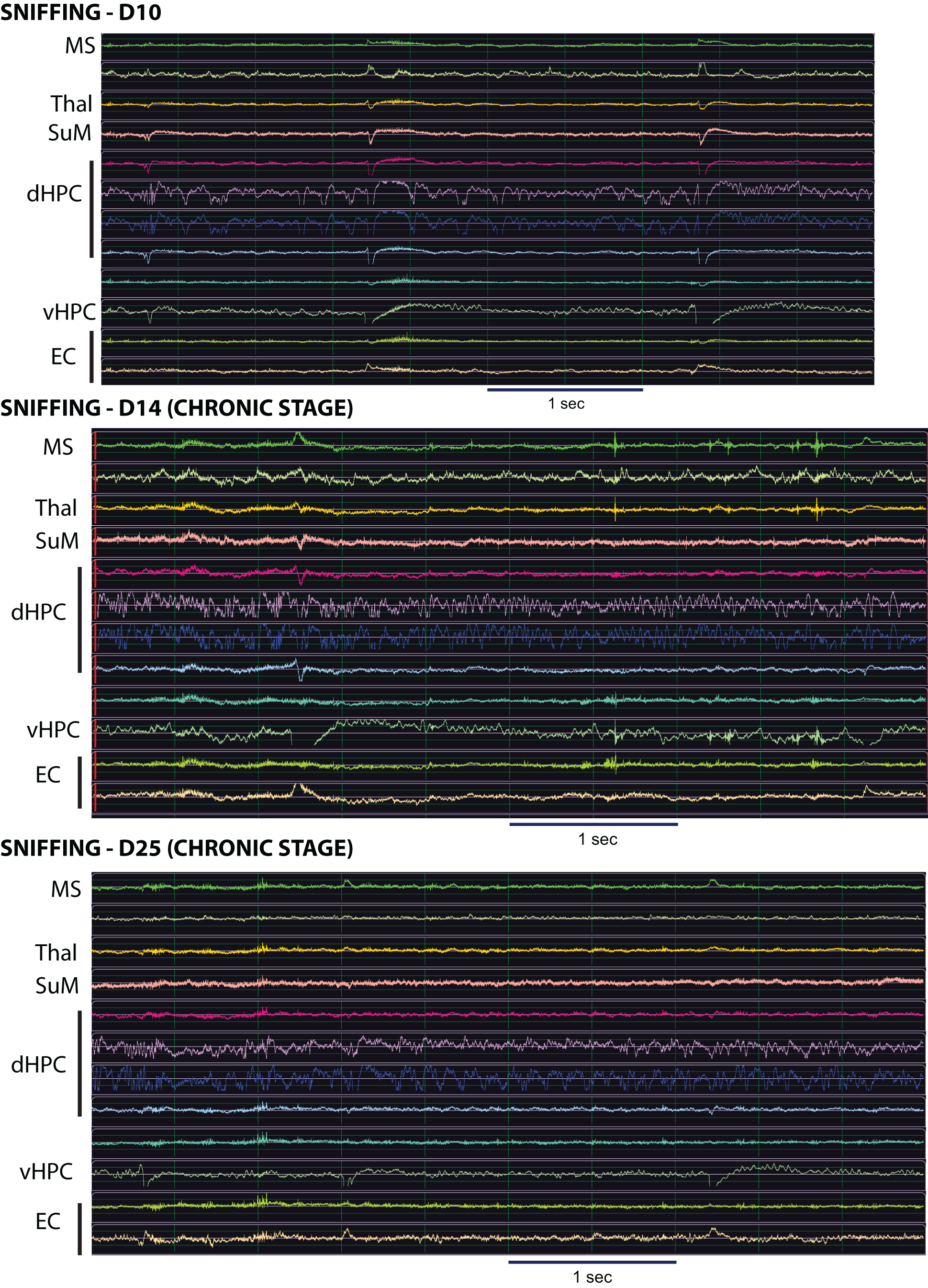

Supplement: Figure 2-1 — Examples of EEG traces. As an extension of Figure 2, this figure depicts examples of EEG traces for each TL brain region considered in the present study at D10, D14, and D25 during sniffing behavior. X axis: time (in sec); Y axis: amplitude (in mV). The scale is shown below each set of traces. Download Figure 2-1, TIF file. [file eneuro-12-ENEURO.0403-24.2025-s004.tif]

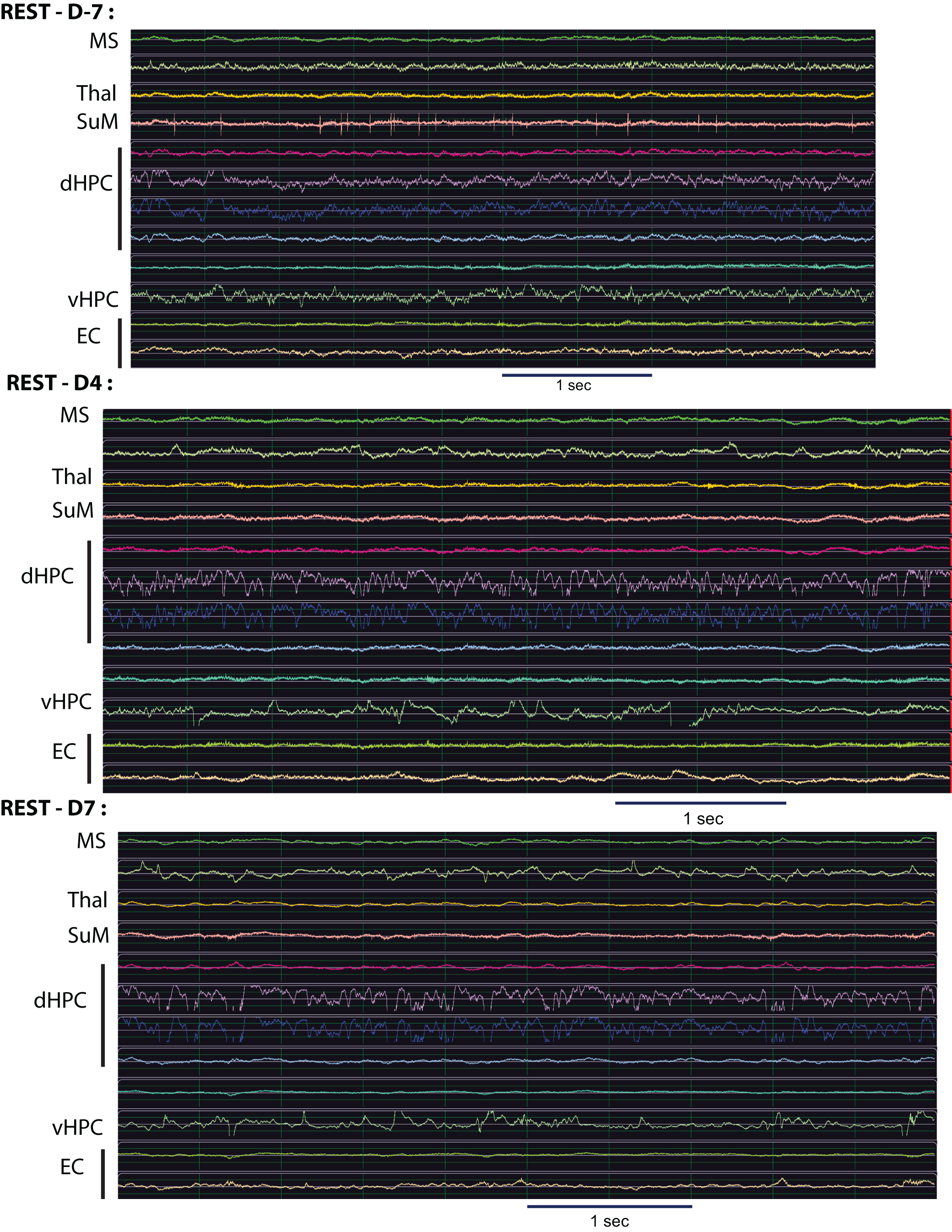

Supplement: Figure 2-2 — Examples of EEG traces. As an extension of Figure 2, this figure depicts examples of EEG traces for each TL brain region considered in the present study at D-7 (control stage), D4, and D7 during rest behavior (awake immobility). X axis: time (in sec); Y axis: amplitude (in mV). The scale is shown below each set of traces. Download Figure 2-2, TIF file. [file eneuro-12-ENEURO.0403-24.2025-s005.tif]

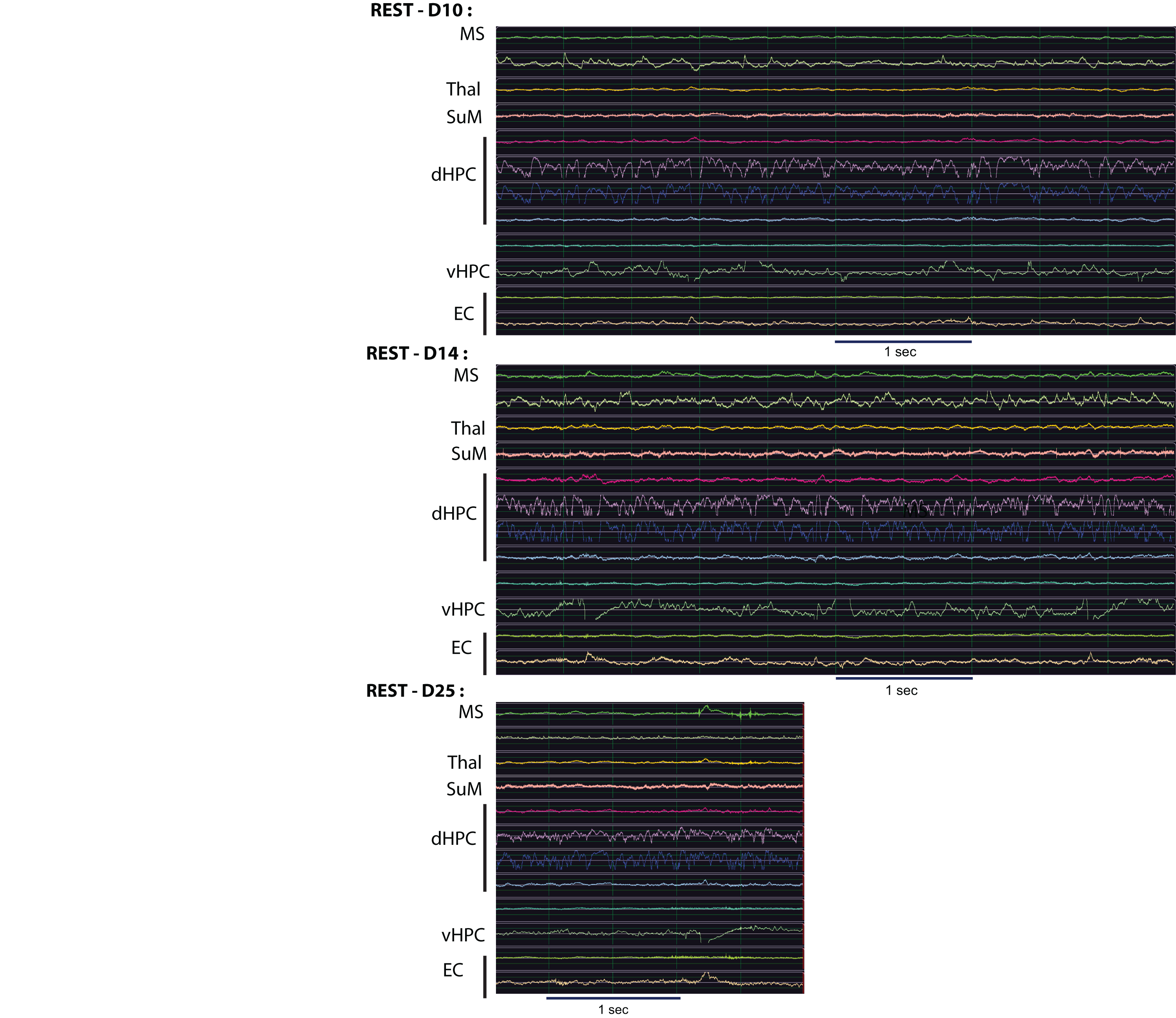

Supplement: Figure 2-3 — Examples of EEG traces. As an extension of Figure 2, this figure depicts examples of EEG traces for each TL brain region considered in the present study at D10, D14, and D25 during rest behavior (awake immobility). X axis: time (in sec); Y axis: amplitude (in mV). The scale is shown below each set of traces. Download Figure 2-3, TIF file. [file eneuro-12-ENEURO.0403-24.2025-s006.tif]

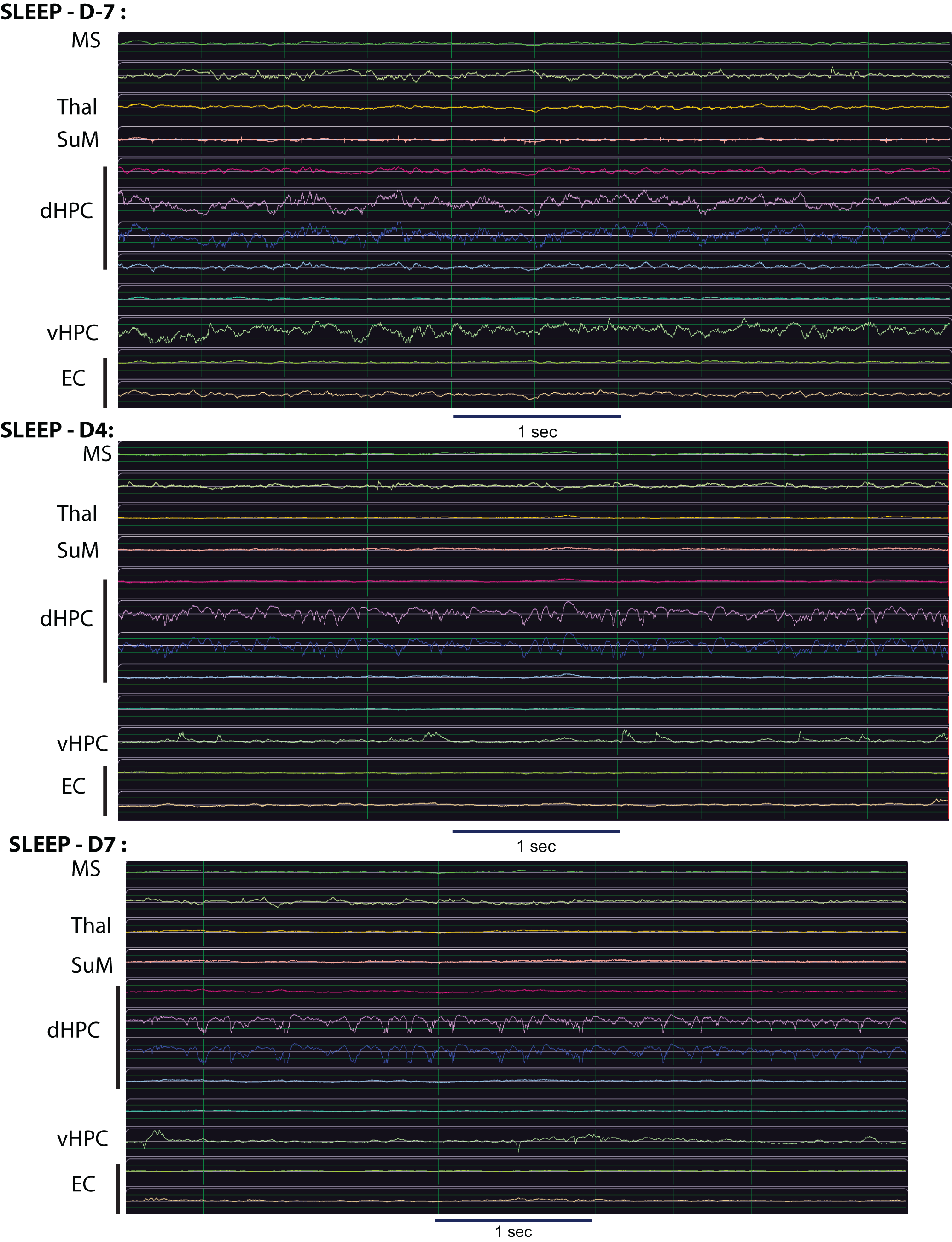

Supplement: Figure 2-4 — Examples of EEG traces. As an extension of Figure 2, this figure depicts examples of EEG traces for each TL brain region considered in the present study at D-7 (control stage), D4, and D7 during sleep behavior (slow wave sleep). X axis: time (in sec); Y axis: amplitude (in mV). The scale is shown below each set of traces. Download Figure 2-4, TIF file. [file eneuro-12-ENEURO.0403-24.2025-s007.tif]

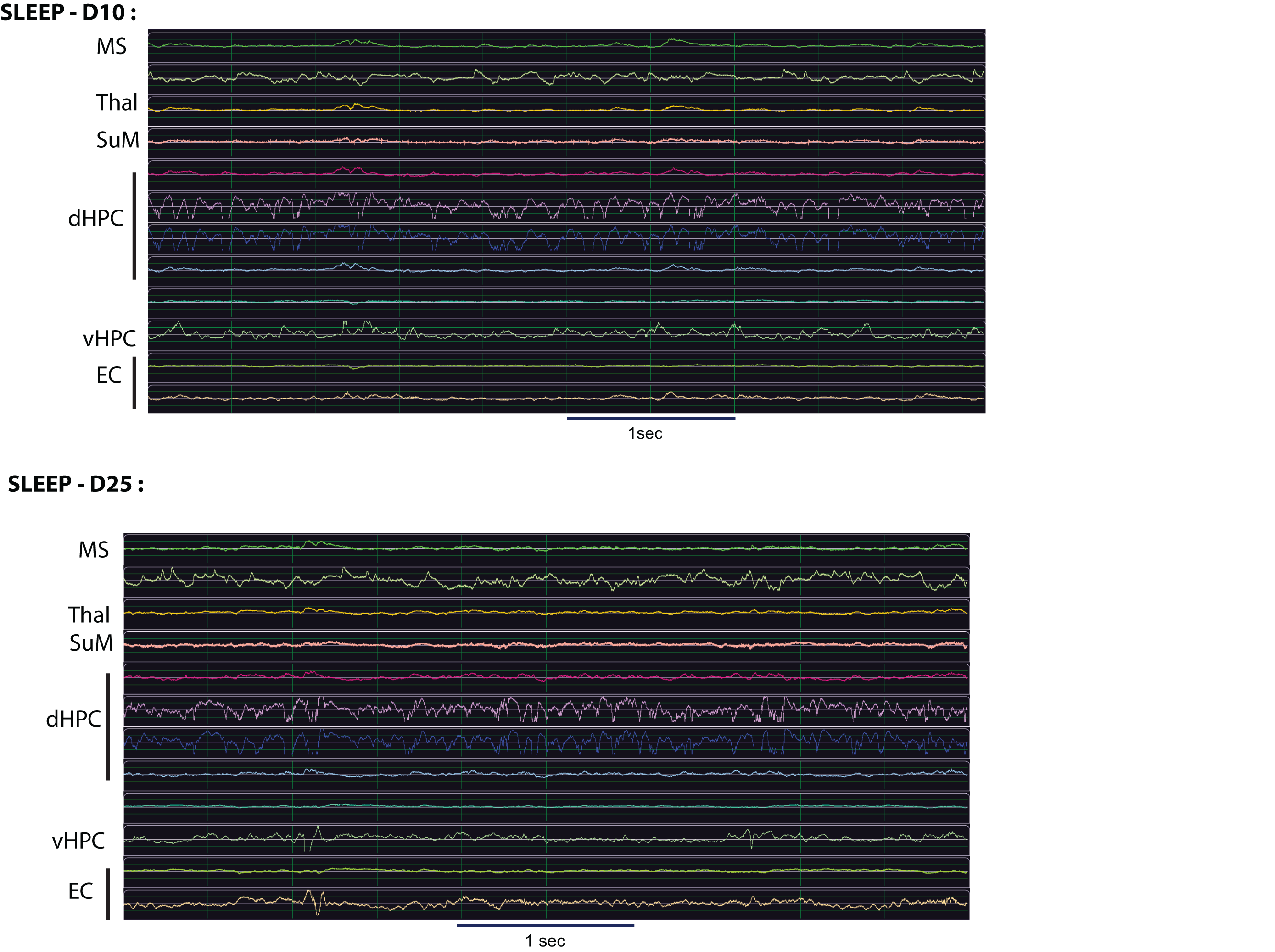

Supplement: Figure 2-5 — Examples of EEG traces. As an extension of Figure 2, this figure depicts examples of EEG traces for each TL brain region considered in the present study at D10 and D25 during sleep behavior (slow wave sleep). Note the absence of epochs at D14 during sleep. X axis: time (in sec); Y axis: amplitude (in mV). The scale is shown below each set of traces. Download Figure 2-5, TIF file. [file eneuro-12-ENEURO.0403-24.2025-s008.tif]
