# Supplementary material for: High-Order Information Analysis of Epileptogenesis in the Pilocarpine Rat Model of Temporal Lobe Epilepsy
Source: eNeuro. 2025 May 21;12(5):ENEURO.0403-24.2025. doi: 10.1523/ENEURO.0403-24.2025 (PMC12121938; doi:10.1523/ENEURO.0403-24.2025)
Supplement: Figure 9-1 — Statistics corresponding to the third section of the Results entitled “Regional distribution of redundant and synergistic multiplets during epileptogenesis”. Download Figure 9-1, DOC file. [file eneuro-12-ENEURO.0403-24.2025-s010.doc]

## **Category A**

### **Sniffing Behavior**

#### **Dorsal Hippocampus (dHPC)**

##### **Redundancy**

| **Time Point** | **p-value** | **Effect Size** |
| --- | --- | --- |
| D4 | <0.001 | 1.418 |
| D7 | 0.029 | 0.338 |
| D10 | 0.002 | 0.394 |
| D14 | 0.903 | 0.100 (negligible) |
| D25 | 0.903 | 0.312 |

##### **Synergy**

| **Time Point** | **p-value** | **Effect Size** |
| --- | --- | --- |
| D4 | <0.001 | 0.889 |
| D7 | 0.011 | 0.376 |
| D10 | 0.011 | 0.186 |
| D14 | 1.000 | 0.287 |
| D25 | 1.000 | 0.167 |

#### **Medial Septum (MS)**

##### **Redundancy**

| **Time Point** | **p-value** | **Effect Size** |
| --- | --- | --- |
| D4 | <0.001 | 1.458 |
| D7 | <0.001 | 0.456 |
| D10 | <0.001 | 0.646 |
| D14 | 0.279 | 0.324 |
| D25 | 0.733 | 0.225 |

##### **Synergy**

| **Time Point** | **p-value** | **Effect Size** |
| --- | --- | --- |
| D4 | <0.001 | 0.903 |
| D7 | <0.001 | 0.528 |
| D10 | <0.001 | 0.358 |
| D14 | 1.000 | 0.011 (negligible) |
| D25 | 1.000 | 0.120 |

#### **Thalamus (Thal)**

##### **Redundancy**

| **Time Point** | **p-value** | **Effect Size** |
| --- | --- | --- |
| D4 | <0.001 | 1.480 |
| D7 | 0.113 | 0.272 |
| D10 | 0.001 | 0.423 |
| D14 | 0.823 | 0.085 (negligible) |
| D25 | (Not specified) | 0.287 |

##### **Synergy**

| **Time Point** | **p-value** | **Effect Size** |
| --- | --- | --- |
| D4 | <0.001 | 0.929 |
| D7 | <0.001 | 0.368 |
| D10 | 0.018 | 0.188 |
| D14 | 1.000 | 0.344 |
| D25 | 1.000 | 0.328 |

### **Rest Behavior**

#### **Medial Septum (MS)**

##### **Redundancy**

| **Time Point** | **p-value** | **Effect Size** |
| --- | --- | --- |
| D4 | <0.001 | 0.712 |
| D7 | 0.032 | 0.422 |
| D10 | <0.001 | 0.566 |
| D14 | 0.014 | 0.905 |
| D25 | 1.000 | 0.011 (negligible) |

##### **Synergy**

| **Time Point** | **p-value** | **Effect Size** |
| --- | --- | --- |
| D4 | 0.121 | 0.351 |
| D7 | 0.040 | 0.429 |
| D10 | 0.205 | 0.342 |
| D14 | 0.205 | 0.628 |
| D25 | (Not specified) | (Not specified) |

#### **Thalamus (Thal)**

##### **Redundancy**

| **Time Point** | **p-value** | **Effect Size** |
| --- | --- | --- |
| D4 | <0.001 | 0.737 |
| D7 | 0.109 | 0.373 |
| D10 | 0.001 | 0.569 |
| D14 | 0.020 | 0.876 |
| D25 | 0.980 | 0.173 |

##### **Synergy**

| **Time Point** | **p-value** | **Effect Size** |
| --- | --- | --- |
| D4 | 0.020 | 0.444 |
| D7 | 0.045 | 0.420 |
| D10 | 0.020 | 0.502 |
| D14 | 0.160 | 0.586 |
| D25 | 0.711 | 0.088 (negligible) |

#### **Dorsal Hippocampus (dHPC)**

##### **Redundancy**

| **Time Point** | **p-value** | **Effect Size** |
| --- | --- | --- |
| D4 | <0.001 | 0.683 |
| D7 | 0.152 | 0.353 |
| D10 | 0.016 | 0.451 |
| D14 | 0.022 | 0.880 |
| D25 | 0.749 | 0.011 (negligible) |

##### **Synergy**

| **Time Point** | **p-value** | **Effect Size** |
| --- | --- | --- |
| No significant changes observed (p > 0.05) |  |  |

#### **Ventral Hippocampus (vHPC)**

##### **Redundancy**

| **Time Point** | **p-value** | **Effect Size** |
| --- | --- | --- |
| D4 | 0.004 | 0.627 |
| D7 | 1.000 | 0.183 |
| D10 | 0.039 | 0.455 |
| D14 | 0.096 | 0.746 |

##### **Synergy**

| **Time Point** | **p-value** | **Effect Size** |
| --- | --- | --- |
| No significant changes observed (p > 0.05) |  |  |

### **Sleep Behavior**

#### **Medial Septum (MS)**

##### **Redundancy**

| **Time Point** | **p-value** | **Effect Size** |
| --- | --- | --- |
| D4 | 0.044 | 1.514 (decrease) |
| D7 | 0.572 | 0.508 |
| D10 | (Not specified) | (Not specified) |

##### **Synergy**

| **Time Point** | **p-value** | **Effect Size** |
| --- | --- | --- |
| D4 | 0.019 | 1.816 (increase) |
| D7 | 0.158 | 0.631 |
| D10 | (Not specified) | (Not specified) |
| D25 | (Not specified) | (Not specified) |

#### **Dorsal Hippocampus (dHPC)**

##### **Redundancy**

| **Time Point** | **p-value** | **Effect Size** |
| --- | --- | --- |
| D4 | 0.038 | 1.851 (decrease) |
| D7 | 0.723 | 0.497 |
| D10 | 0.006 | 0.714 (decrease) |
| D25 | (Not specified) | (Not specified) |

##### **Synergy**

| **Time Point** | **p-value** | **Effect Size** |
| --- | --- | --- |
| D4 | 0.016 | 1.992 (increase) |
| D7 | 0.261 | 0.579 |
| D10 | 0.007 | 0.845 |
| D25 | 0.050 | 1.462 |

#### **Thalamus (Thal)**

##### **Redundancy**

| **Time Point** | **p-value** | **Effect Size** |
| --- | --- | --- |
| D10 | 0.056 | 0.117 (not significant) |
| D25 | 0.012 | 2.107 |

##### **Synergy**

| **Time Point** | **p-value** | **Effect Size** |
| --- | --- | --- |
| D4 | 0.023 | 1.115 (increase) |
| D7 | 0.308 | 0.516 |
| D10 | 0.011 | 0.297 |
| D25 | 0.008 | 1.643 |

#### **Ventral Hippocampus (vHPC)**

##### **Redundancy**

| **Time Point** | **p-value** | **Effect Size** |
| --- | --- | --- |
| D10 | 0.003 | 0.756 |
| D25 | 0.029 | 1.914 |

##### **Synergy**

| **Time Point** | **p-value** | **Effect Size** |
| --- | --- | --- |
| D10 | 0.006 | 0.949 |
| D25 | 0.048 | 1.736 |

## **Category B**

### **Sniffing Behavior**

#### **Dorsal Hippocampus (dHPC)**

##### **Redundancy**

| **Time Point** | **p-value** | **Effect Size** |
| --- | --- | --- |
| D4 | <0.001 | 1.042 |
| D7 | <0.001 | 0.899 |
| D10 | <0.001 | 0.977 |
| D14 | <0.001 | 1.312 |
| D25 | 1.000 | 0.087 (negligible) |

##### **Synergy**

| **Time Point** | **p-value** | **Effect Size** |
| --- | --- | --- |
| D4 | <0.001 | 0.598 |
| D7 | <0.001 | 0.785 |
| D10 | <0.001 | 0.843 |
| D14 | <0.001 | 1.001 |
| D25 | 1.000 | 0.093 (negligible) |

#### **Medial Septum (MS)**

##### **Redundancy**

| **Time Point** | **p-value** | **Effect Size** |
| --- | --- | --- |
| D4 | <0.001 | 1.016 |
| D7 | <0.001 | 1.064 |
| D10 | <0.001 | 1.038 |
| D14 | <0.001 | 1.305 |
| D25 | 1.000 | 0.044 (negligible) |

##### **Synergy**

| **Time Point** | **p-value** | **Effect Size** |
| --- | --- | --- |
| D4 | <0.001 | 0.472 |
| D7 | <0.001 | 0.870 |
| D10 | <0.001 | 0.755 |
| D14 | <0.001 | 0.925 |
| D25 | 1.000 | 0.183 |

#### **Supramammillary Nucleus (SuM)**

##### **Redundancy**

| **Time Point** | **p-value** | **Effect Size** |
| --- | --- | --- |
| D4 | <0.001 | 1.074 |
| D7 | <0.001 | 1.060 |
| D10 | <0.001 | 1.037 |
| D14 | <0.001 | 1.382 |
| D25 | 1.000 | 0.074 (negligible) |

##### **Synergy**

| **Time Point** | **p-value** | **Effect Size** |
| --- | --- | --- |
| D4 | <0.001 | 0.561 |
| D7 | <0.001 | 1.016 |
| D10 | <0.001 | 0.862 |
| D14 | <0.001 | 0.988 |
| D25 | 1.000 | 0.122 (negligible) |

#### **Entorhinal Cortex (EC)**

##### **Redundancy**

| **Time Point** | **p-value** | **Effect Size** |
| --- | --- | --- |
| D4 | <0.001 | 0.850 |
| D7 | <0.001 | 0.656 |
| D10 | <0.001 | 0.879 |
| D14 | <0.001 | 1.186 |
| D25 | 1.000 | 0.114 (negligible) |

##### **Synergy**

| **Time Point** | **p-value** | **Effect Size** |
| --- | --- | --- |
| D7 | <0.001 | 0.522 |
| D10 | <0.001 | 0.666 |
| D14 | <0.001 | 0.809 |
| D25 | 0.883 | 0.000 (negligible) |

### **Rest Behavior**

#### **Medial Septum (MS)**

##### **Redundancy**

| **Time Point** | **p-value** | **Effect Size** |
| --- | --- | --- |
| D10 | 0.022 | 0.437 |
| D14 | 0.023 | 0.967 |
| D25 | >0.05 | Negligible |

##### **Synergy**

| **Time Point** | **p-value** | **Effect Size** |
| --- | --- | --- |
| No significant changes observed (p > 0.05) |  |  |

#### **Supramammillary Nucleus (SuM)**

##### **Redundancy**

| **Time Point** | **p-value** | **Effect Size** |
| --- | --- | --- |
| D10 | 0.001 | 0.563 |
| D14 | 0.014 | 0.969 |
| D25 | >0.05 | Negligible |

##### **Synergy**

| **Time Point** | **p-value** | **Effect Size** |
| --- | --- | --- |
| No significant changes observed (p > 0.05) |  |  |

#### **Dorsal Hippocampus (dHPC)**

##### **Redundancy**

| **Time Point** | **p-value** | **Effect Size** |
| --- | --- | --- |
| D10 | 0.006 | 0.481 |
| D14 | 0.020 | 0.956 |
| D25 | >0.05 | Negligible |

##### **Synergy**

| **Time Point** | **p-value** | **Effect Size** |
| --- | --- | --- |
| D7 | 0.009 | 0.446 |
| D10 | 0.128 | 0.173 (not significant) |
| D25 | 0.150 | 0.311 |

#### **Entorhinal Cortex (EC)**

##### **Redundancy**

| **Time Point** | **p-value** | **Effect Size** |
| --- | --- | --- |
| D14 | 0.015 | 1.012 |
| D25 | 1.000 | 0.287 (normalized) |

##### **Synergy**

| **Time Point** | **p-value** | **Effect Size** |
| --- | --- | --- |
| D4 | 0.032 | 0.378 |
| D7 | 1.000 | 0.084 (negligible) |
| D10 | 1.000 | 0.033 (negligible) |
| D25 | 1.000 | 0.234 (negligible) |

### **Sleep Behavior**

#### **All Brain Regions**

##### **Redundancy**

| **Brain Region** | **Time Point** | **p-value** | **Effect Size** |
| --- | --- | --- | --- |
| Entorhinal Cortex (EC) | D10 | 0.004 | 0.943 |
| Medial Septum (MS) | D10 | 0.011 | 0.580 |
| Dorsal Hippocampus (dHPC) | D10 | 0.035 | 0.385 |
| Supramammillary Nucleus (SuM) | D10 | 0.037 | 0.397 |

##### **Synergy**

- **No significant changes observed (p > 0.05) for all brain regions.**
