# Supplementary material for: High-Order Information Analysis of Epileptogenesis in the Pilocarpine Rat Model of Temporal Lobe Epilepsy
Source: eNeuro. 2025 May 21;12(5):ENEURO.0403-24.2025. doi: 10.1523/ENEURO.0403-24.2025 (PMC12121938; doi:10.1523/ENEURO.0403-24.2025)
Supplement: Figure 10-1 — Statistics corresponding to the fourth section of the Results entitled “Evolution of triplets and quadruplets during epileptogenesis”. Download Figure 10-1, DOC file. [file eneuro-12-ENEURO.0403-24.2025-s011.doc]

### **Category A**

#### **Sniffing Behavior**

##### **Redundancy**

**Triplet: MS-Thal-dHPC**

| **Time Point** | **p-value** | **Effect Size** |
| --- | --- | --- |
| D4 | <0.001 | 1.531 |
| D7 | <0.001 | 0.672 |
| D10 | <0.001 | 0.518 |
| D14 | 0.339 | 0.334 |
| D25 | 0.714 | 0.088 (negligible) |

**Triplet: MS-Thal-vHPC**

| **Time Point** | **p-value** | **Effect Size** |
| --- | --- | --- |
| D4 | 0.002 | 0.582 |
| D7 | 0.946 | 0.127 |
| D10 | 0.027 | 0.329 |
| D14 | 0.947 | 0.124 |
| D25 | 0.946 | 0.278 |

**Triplet: MS-dHPC-vHPC**

| **Time Point** | **p-value** | **Effect Size** |
| --- | --- | --- |
| D4 | 0.009 | 0.515 |
| D7 | 1.000 | 0.045 (negligible) |
| D10 | 0.181 | 0.256 |
| D14 | 1.000 | 0.171 |
| D25 | 0.704 | 0.358 |

**Triplet: Thal-dHPC-vHPC**

| **Time Point** | **p-value** | **Effect Size** |
| --- | --- | --- |
| D7 | <0.001 | 0.430 |
| D10 | 0.016 | 0.337 |
| D14 | 0.050 | 0.510 |
| D25 | 0.254 | 0.500 |

**Quadruplet: MS-Thal-dHPC-vHPC**

| **Time Point** | **p-value** | **Effect Size** |
| --- | --- | --- |
| D4 | <0.001 | 1.475 |
| D7 | <0.001 | 0.673 |
| D10 | <0.001 | 0.760 |
| D14 | 0.380 | 0.298 |
| D25 | 1.000 | 0.097 (negligible) |

##### **Synergy**

**Triplet: MS-Thal-dHPC**

| **Time Point** | **p-value** | **Effect Size** |
| --- | --- | --- |
| D4 | <0.001 | 1.203 |
| D7 | <0.001 | 0.722 |
| D10 | <0.001 | 0.559 |

**Quadruplet: MS-Thal-dHPC-vHPC**

| **Time Point** | **p-value** | **Effect Size** |
| --- | --- | --- |
| D4 | 0.040 | 0.423 |
| D7 | 0.186 | 0.237 |
| D10 | 1.000 | 0.108 |
| D14 | 0.005 | 0.537 |
| D25 | 1.000 | 0.298 |

#### **Rest Behavior**

##### **Redundancy**

**Triplet: MS-Thal-dHPC**

| **Time Point** | **p-value** | **Effect Size** |
| --- | --- | --- |
| D4 | <0.001 | 0.668 |
| D7 | <0.001 | 0.697 |
| D10 | <0.001 | 0.544 |
| D14 | 0.005 | 1.007 |
| D25 | 0.176 | 0.568 |

**Quadruplet: MS-Thal-dHPC-vHPC**

| **Time Point** | **p-value** | **Effect Size** |
| --- | --- | --- |
| D4 | <0.001 | 0.772 |
| D7 | 0.023 | 0.377 |
| D10 | 0.002 | 0.534 |
| D14 | 0.289 | 0.658 |
| D25 | 1.000 | 0.383 |

##### **Synergy**

**Triplet: MS-Thal-dHPC**

| **Time Point** | **p-value** | **Effect Size** |
| --- | --- | --- |
| D4 | 0.019 | 0.496 |
| D7 | <0.001 | 0.526 |
| D10 | 0.010 | 0.445 |
| D14 | 0.066 | 0.745 |
| D25 | 0.121 | 0.571 |

**Quadruplet: MS-Thal-dHPC-vHPC**

| **Time Point** | **p-value** | **Effect Size** |
| --- | --- | --- |
| D4 | 0.412 | 0.285 |
| D7 | 0.005 | 0.420 |
| D10 | 0.158 | 0.291 |
| D14 | 1.000 | 0.349 |
| D25 | 0.958 | 0.354 |

#### **Sleep Behavior**

##### **Redundancy**

**Triplet: MS-Thal-dHPC**

| **Time Point** | **p-value** | **Effect Size** |
| --- | --- | --- |
| D4 | <0.001 | 2.500 |
| D7 | 0.306 | 0.481 |
| D10 | 0.306 | 0.481 |
| D25 | 0.301 | 0.981 |

**Triplet: MS-dHPC-vHPC**

| **Time Point** | **p-value** | **Effect Size** |
| --- | --- | --- |
| D10 | <0.001 | 1.437 |

**Triplet: Thal-dHPC-vHPC**

| **Time Point** | **p-value** | **Effect Size** |
| --- | --- | --- |
| D10 | <0.001 | 1.487 |
| D25 | 0.004 | 2.944 |

##### **Synergy**

**Triplet: MS-Thal-dHPC**

| **Time Point** | **p-value** | **Effect Size** |
| --- | --- | --- |
| D4 | <0.001 | 2.944 |
| D7 | 0.512 | 0.409 |
| D10 | 0.512 | 0.409 |
| D25 | 0.121 | 1.196 |

**Triplet: MS-dHPC-vHPC**

| **Time Point** | **p-value** | **Effect Size** |
| --- | --- | --- |
| D4 | 0.011 | 2.500 |
| D7 | 0.818 | 0.481 |
| D10 | <0.001 | 1.316 |
| D25 | 0.818 | 0.981 |

**Triplet: Thal-dHPC-vHPC**

| **Time Point** | **p-value** | **Effect Size** |
| --- | --- | --- |
| D10 | <0.001 | 1.487 |
| D25 | 0.004 | 2.944 |

### **Category B**

#### **Sniffing Behavior**

##### **Redundancy**

**Triplet: EC-MS-SuM**

| **Time Point** | **p-value** | **Effect Size** |
| --- | --- | --- |
| D4 | <0.001 | 0.566 |
| D7 | <0.001 | 0.673 |
| D10 | <0.001 | 0.660 |
| D14 | <0.001 | 0.877 |
| D25 | 0.240 | 0.444 |

**Quadruplet: EC-MS-SuM-dHPC**

| **Time Point** | **p-value** | **Effect Size** |
| --- | --- | --- |
| D4 | <0.001 | 0.923 |
| D7 | <0.001 | 0.919 |
| D10 | <0.001 | 1.041 |
| D14 | <0.001 | 1.222 |
| D25 | 1.000 | 0.032 (negligible) |

**Triplet: EC-MS-dHPC**

| **Time Point** | **p-value** | **Effect Size** |
| --- | --- | --- |
| D4 | <0.001 | 0.577 |
| D7 | 0.005 | 0.345 |
| D10 | <0.001 | 0.607 |
| D14 | <0.001 | 0.824 |
| D25 | 1.000 | 0.070 (negligible) |

**Triplet: MS-SuM-dHPC**

| **Time Point** | **p-value** | **Effect Size** |
| --- | --- | --- |
| D4 | <0.001 | 1.294 |
| D7 | <0.001 | 1.682 |
| D10 | <0.001 | 1.063 |
| D14 | <0.001 | 1.410 |
| D25 | 0.954 | 0.216 |

**Triplet: EC-SuM-dHPC**

| **Time Point** | **p-value** | **Effect Size** |
| --- | --- | --- |
| D7 | 0.088 | 0.253 |

##### **Synergy**

**Triplet: EC-SuM-dHPC**

| **Time Point** | **p-value** | **Effect Size** |
| --- | --- | --- |
| D4 | 0.015 | 0.399 |
| D7 | <0.001 | 0.366 |
| D10 | <0.001 | 0.570 |
| D14 | <0.001 | 0.691 |
| D25 | 0.855 | 0.313 |

**Triplet: MS-SuM-dHPC**

| **Time Point** | **p-value** | **Effect Size** |
| --- | --- | --- |
| D4 | <0.001 | 0.631 |
| D7 | <0.001 | 1.137 |
| D10 | <0.001 | 0.746 |
| D14 | <0.001 | 0.870 |
| D25 | 1.000 | 0.141 |

**Triplet: EC-MS-SuM**

| **Time Point** | **p-value** | **Effect Size** |
| --- | --- | --- |
| D7 | <0.001 | 0.459 |
| D10 | 0.005 | 0.351 |
| D14 | 0.073 | 0.418 |
| D25 | 0.164 | 0.425 |

**Quadruplet: EC-MS-SuM-dHPC**

| **Time Point** | **p-value** | **Effect Size** |
| --- | --- | --- |
| D7 | <0.001 | 0.518 |
| D10 | <0.001 | 0.429 |
| D14 | 0.017 | 0.409 |

#### **Rest Behavior**

##### **Redundancy**

**Triplet: MS-SuM-dHPC**

| **Time Point** | **p-value** | **Effect Size** |
| --- | --- | --- |
| D4 | 0.007 | 0.520 |
| D7 | <0.001 | 0.757 |
| D10 | <0.001 | 0.569 |
| D14 | 0.204 | 0.595 |

##### **Synergy**

**Triplet: MS-SuM-dHPC**

| **Time Point** | **p-value** | **Effect Size** |
| --- | --- | --- |
| D7 | <0.001 | 0.685 |
| D10 | 0.005 | 0.439 |
| D14 | 0.208 | 0.593 |
| D25 | 0.669 | 0.406 |

#### **Sleep Behavior**

##### **Redundancy**

**Triplet: MS-SuM-dHPC**

| **Time Point** | **p-value** | **Effect Size** |
| --- | --- | --- |
| D4 | 0.005 | 1.958 |
| D7 | 0.220 | 0.615 |
| D10 | 0.220 | 0.615 |
| D25 | 0.376 | 0.688 |

**Triplet: EC-MS-dHPC**

| **Time Point** | **p-value** | **Effect Size** |
| --- | --- | --- |
| D10 | <0.001 | 1.187 |

**Triplet: EC-MS-SuM**

| **Time Point** | **p-value** | **Effect Size** |
| --- | --- | --- |
| D10 | 0.002 | 1.230 |

##### **Synergy**

**Triplet: MS-SuM-dHPC**

| **Time Point** | **p-value** | **Effect Size** |
| --- | --- | --- |
| D4 | 0.005 | 1.958 |
| D7 | 0.220 | 0.615 |
| D10 | 0.220 | 0.615 |
| D25 | 0.376 | 0.688 |

**Triplet: EC-MS-dHPC**

| **Time Point** | **p-value** | **Effect Size** |
| --- | --- | --- |
| D10 | 0.027 | 0.838 |
