# Supplementary material for: High-Order Information Analysis of Epileptogenesis in the Pilocarpine Rat Model of Temporal Lobe Epilepsy
Source: eNeuro. 2025 May 21;12(5):ENEURO.0403-24.2025. doi: 10.1523/ENEURO.0403-24.2025 (PMC12121938; doi:10.1523/ENEURO.0403-24.2025)
Supplement: Figure 11-1 — Statistics corresponding to the fifth section of the Results entitled “Dynamics of O-information along epileptogenesis”. Download Figure 11-1, DOC file. [file eneuro-12-ENEURO.0403-24.2025-s012.doc]

## **Category A**

### **Sniffing Behavior**

#### **Redundant Triplets**

**Overall O-Information**

| **Time Point** | **p-value** | **Effect Size** |
| --- | --- | --- |
| **D4** | <0.001 | 1.209 |
| **D7** | 0.002 | 0.539 |
| **D10** | <0.001 | 0.072 (negligible) |
| **D14** | 0.899 | 0.119 |
| **D25** | 0.657 | 0.113 |

**Per Brain Region**

- **Medial Septum (MS)**

| **Time Point** | **p-value** | **Effect Size** |
| --- | --- | --- |
| D4 | <0.001 | 1.192 |
| D7 | 0.008 | 1.192 |
| D10 | <0.001 | 0.095 (negligible) |
| D14 | 1.000 | 0.119 |
| D25 | 1.000 | 0.104 |

- **Thalamus (Thal)**

| **Time Point** | **p-value** | **Effect Size** |
| --- | --- | --- |
| D4 | <0.001 | 1.189 |
| D7 | 0.009 | 0.569 |
| D10 | <0.001 | 0.130 |
| D14 | 1.000 | 0.149 |
| D25 | 1.000 | 0.116 |

- **Dorsal Hippocampus (dHPC)**

| **Time Point** | **p-value** | **Effect Size** |
| --- | --- | --- |
| D4 | <0.001 | 1.116 |
| D7 | 0.090 | 0.507 |
| D10 | 0.031 | 0.087 (negligible) |
| D14 | 0.199 | 0.113 |
| D25 | 0.901 | 0.100 |

- **Ventral Hippocampus (vHPC)**

| **Time Point** | **p-value** | **Effect Size** |
| --- | --- | --- |
| D4 | <0.001 | 1.331 |
| D7 | 0.007 | 0.584 |
| D10 | <0.001 | 0.133 |
| D14 | 1.000 | 0.103 |
| D25 | 0.833 | 0.128 |

#### **Synergistic Triplets**

**Overall O-Information**

| **Time Point** | **p-value** | **Effect Size** |
| --- | --- | --- |
| **D7** | <0.001 | 1.223 |
| **D10** | 0.002 | 0.205 |
| **D14** | <0.001 | 0.451 |
| **D25** | 0.036 | 0.542 |

**Per Brain Region**

- **Medial Septum (MS)**

| **Time Point** | **p-value** | **Effect Size** |
| --- | --- | --- |
| D7 | <0.001 | 1.231 |
| D10 | 0.650 | 0.050 (negligible) |
| D14 | 1.000 | 0.098 (negligible) |
| D25 | 0.074 | 0.594 |

- **Thalamus (Thal)**

| **Time Point** | **p-value** | **Effect Size** |
| --- | --- | --- |
| D7 | <0.001 | 1.211 |
| D10 | <0.001 | 0.264 |
| D14 | <0.001 | 0.470 |
| D25 | 0.029 | 0.539 |

- **Dorsal Hippocampus (dHPC)**

| **Time Point** | **p-value** | **Effect Size** |
| --- | --- | --- |
| D7 | <0.001 | 1.190 |
| D10 | 0.002 | 0.229 |
| D14 | 0.002 | 0.487 |
| D25 | 0.009 | 0.639 |

- **Ventral Hippocampus (vHPC)**

| **Time Point** | **p-value** | **Effect Size** |
| --- | --- | --- |
| D7 | <0.001 | 1.391 |
| D10 | 0.035 | 0.310 |
| D14 | <0.001 | 0.691 |
| D25 | 1.000 | 0.499 |

#### **Redundant Quadruplets**

| **Time Point** | **p-value** | **Effect Size** |
| --- | --- | --- |
| D7 | 0.003 | 0.262 |
| D10 | 1.000 | 0.083 (normalized) |

#### **Synergistic Quadruplets**

- **No significant changes observed across all behaviors (p > 0.05).**

### **Rest Behavior**

#### **Redundant Triplets**

**Overall O-Information**

| **Time Point** | **p-value** | **Effect Size** |
| --- | --- | --- |
| **D7** | 0.006 | 0.662 |
| **D10** | <0.001 | 0.318 |
| **D14** | <0.001 | 0.609 |

**Per Brain Region**

- **Medial Septum (MS)**

| **Time Point** | **p-value** | **Effect Size** |
| --- | --- | --- |
| D7 | 0.003 | 0.658 |
| D10 | <0.001 | 0.526 |
| D14 | <0.001 | 1.028 |
| D25 | 1.000 | 0.258 |

- **Thalamus (Thal)**

| **Time Point** | **p-value** | **Effect Size** |
| --- | --- | --- |
| D7 | 0.012 | 0.656 |
| D10 | 0.003 | 0.297 |
| D14 | <0.001 | 0.550 |
| D25 | 0.855 | 0.327 |

- **Dorsal Hippocampus (dHPC)**

| **Time Point** | **p-value** | **Effect Size** |
| --- | --- | --- |
| D7 | 0.006 | 0.661 |
| D10 | <0.001 | 0.229 |
| D14 | <0.001 | 0.484 |
| D25 | 0.018 | 0.493 |

- **Ventral Hippocampus (vHPC)**

| **Time Point** | **p-value** | **Effect Size** |
| --- | --- | --- |
| D10 | 0.009 | 0.255 |
| D14 | <0.001 | 0.520 |
| D25 | 0.447 | 0.348 |

#### **Synergistic Triplets**

**Overall O-Information**

| **Time Point** | **p-value** | **Effect Size** |
| --- | --- | --- |
| **D7** | <0.001 | 0.910 (decrease) |
| **D10** | <0.001 | 0.592 (decrease) |

**Per Brain Region**

- **Medial Septum (MS)**

| **Time Point** | **p-value** | **Effect Size** |
| --- | --- | --- |
| D7 | <0.001 | 0.808 |
| D10 | <0.001 | 0.547 |

- **Thalamus (Thal)**

| **Time Point** | **p-value** | **Effect Size** |
| --- | --- | --- |
| D7 | <0.001 | 0.950 |
| D10 | 0.009 | 0.481 |

- **Dorsal Hippocampus (dHPC)**

| **Time Point** | **p-value** | **Effect Size** |
| --- | --- | --- |
| D7 | <0.001 | 0.824 |
| D10 | <0.001 | 0.728 |

- **Ventral Hippocampus (vHPC)**

| **Time Point** | **p-value** | **Effect Size** |
| --- | --- | --- |
| D7 | <0.001 | 1.059 |
| D10 | 0.001 | 0.609 |

### **Sleep Behavior**

#### **Redundant Triplets**

**Overall O-Information**

| **Time Point** | **p-value** | **Effect Size** |
| --- | --- | --- |
| **D7** | <0.001 | 1.180 |
| **D10** | <0.001 | 0.947 |

**Per Brain Region**

- **Medial Septum (MS)**

| **Time Point** | **p-value** | **Effect Size** |
| --- | --- | --- |
| D7 | <0.001 | 1.226 |
| D10 | <0.001 | 0.963 |

- **Thalamus (Thal)**

| **Time Point** | **p-value** | **Effect Size** |
| --- | --- | --- |
| D7 | <0.001 | 1.130 |
| D10 | <0.001 | 1.112 |

- **Dorsal Hippocampus (dHPC)**

| **Time Point** | **p-value** | **Effect Size** |
| --- | --- | --- |
| D7 | <0.001 | 1.197 |
| D10 | 0.006 | 0.895 |

- **Ventral Hippocampus (vHPC)**

| **Time Point** | **p-value** | **Effect Size** |
| --- | --- | --- |
| D7 | <0.001 | 1.160 |
| D10 | 0.003 | 0.862 |

#### **Synergistic Triplets**

- **No significant changes observed (p > 0.05).**

#### **Redundant Quadruplets**

| **Time Point** | **p-value** | **Effect Size** |
| --- | --- | --- |
| **D10** | <0.001 | 2.231 |

## **Category B**

### **Sniffing Behavior**

#### **Redundant Triplets**

**Overall O-Information**

| **Time Point** | **p-value** | **Effect Size** |
| --- | --- | --- |
| **D4** | <0.001 | 0.452 |

**Per Brain Region**

- **Entorhinal Cortex (EC)**

| **Time Point** | **p-value** | **Effect Size** |
| --- | --- | --- |
| D4 | <0.001 | 0.386 |
| D7 | 1.000 | 1.192 |

- **Medial Septum (MS)**

| **Time Point** | **p-value** | **Effect Size** |
| --- | --- | --- |
| D4 | <0.001 | 0.596 |
| D7 | 1.000 | 0.209 |

- **Supramammillary Nucleus (SuM)**

| **Time Point** | **p-value** | **Effect Size** |
| --- | --- | --- |
| D4 | <0.001 | 0.414 |
| D7 | 0.008 | 0.057 (negligible) |

- **Dorsal Hippocampus (dHPC)**

| **Time Point** | **p-value** | **Effect Size** |
| --- | --- | --- |
| D4 | <0.001 | 0.418 |
| D7 | 0.005 | 0.083 (negligible) |

#### **Synergistic Triplets**

**Overall O-Information**

| **Time Point** | **p-value** | **Effect Size** |
| --- | --- | --- |
| **D4** | <0.001 | 0.598 |
| **D7** | <0.001 | 0.675 |
| **D10** | 1.000 | 0.120 (normalized) |

**Per Brain Region**

- **Entorhinal Cortex (EC)**

| **Time Point** | **p-value** | **Effect Size** |
| --- | --- | --- |
| D4 | <0.001 | 0.816 |
| D7 | <0.001 | 1.418 |
| D10 | <0.001 | 1.177 |
| D25 | 1.000 | 0.225 |

- **Medial Septum (MS)**

| **Time Point** | **p-value** | **Effect Size** |
| --- | --- | --- |
| D4 | <0.001 | 0.862 |
| D7 | <0.001 | 1.302 |
| D10 | <0.001 | 1.127 |
| D25 | 1.000 | 0.286 |

- **Supramammillary Nucleus (SuM)**

| **Time Point** | **p-value** | **Effect Size** |
| --- | --- | --- |
| D4 | 0.001 | 0.786 |
| D7 | <0.001 | 1.362 |
| D10 | <0.001 | 1.005 |
| D25 | 1.000 | 1.057 |

- **Dorsal Hippocampus (dHPC)**

| **Time Point** | **p-value** | **Effect Size** |
| --- | --- | --- |
| D4 | <0.001 | 0.964 |
| D7 | <0.001 | 1.491 |
| D10 | <0.001 | 1.204 |
| D25 | 0.420 | 1.115 |

#### **Redundant Quadruplets**

| **Time Point** | **p-value** | **Effect Size** |
| --- | --- | --- |
| **D7** | <0.001 | 0.141 (trivial) |
| **D10** | <0.001 | 0.725 |
| **D14** | 0.014 | 0.598 |
| **D25** | 0.997 | 0.315 |

#### **Synergistic Quadruplets**

- **No significant changes observed across all behaviors (p > 0.05).**

### **Rest Behavior**

#### **Redundant Triplets**

**Overall O-Information**

| **Time Point** | **p-value** | **Effect Size** |
| --- | --- | --- |
| **D4** | 0.024 | 0.174 (trivial decrease) |
| **D7** | <0.001 | 0.530 |
| **D10** | <0.001 | 0.004 (negligible) |
| **D14** | <0.001 | 0.104 (small) |
| **D25** | <0.001 | 0.514 |

**Per Brain Region**

- **Entorhinal Cortex (EC)**

| **Time Point** | **p-value** | **Effect Size** |
| --- | --- | --- |
| D4 | 0.001 | 0.151 |
| D7 | <0.001 | 0.569 |
| D10 | <0.001 | 0.017 (negligible) |
| D14 | <0.001 | 0.080 (negligible) |
| D25 | 0.002 | 0.472 |

- **Medial Septum (MS)**

| **Time Point** | **p-value** | **Effect Size** |
| --- | --- | --- |
| D4 | 0.048 | 0.306 |
| D7 | <0.001 | 0.586 |
| D10 | <0.001 | 0.156 (small) |
| D14 | <0.001 | 0.127 (small) |
| D25 | 0.002 | 0.455 |

- **Supramammillary Nucleus (SuM)**

| **Time Point** | **p-value** | **Effect Size** |
| --- | --- | --- |
| D7 | 0.006 | 0.486 |
| D10 | 0.011 | 0.038 (negligible) |
| D14 | 0.011 | 0.088 (negligible) |
| D25 | <0.001 | 0.548 |

- **Dorsal Hippocampus (dHPC)**

| **Time Point** | **p-value** | **Effect Size** |
| --- | --- | --- |
| D7 | 0.038 | 0.486 (negligible) |
| D14 | 0.009 | 0.130 (small) |
| D25 | <0.001 | 0.586 |

#### **Synergistic Triplets**

**Overall O-Information**

| **Time Point** | **p-value** | **Effect Size** |
| --- | --- | --- |
| **D4** | <0.001 | 0.851 |
| **D7** | <0.001 | 1.392 |
| **D10** | <0.001 | 1.129 |

### **Sleep Behavior**

#### **Redundant Triplets**

**Overall O-Information**

| **Time Point** | **p-value** | **Effect Size** |
| --- | --- | --- |
| **D7** | <0.001 | 1.015 |

**Per Brain Region**

- **Entorhinal Cortex (EC)**

| **Time Point** | **p-value** | **Effect Size** |
| --- | --- | --- |
| D7 | <0.001 | 1.128 |

- **Medial Septum (MS)**

| **Time Point** | **p-value** | **Effect Size** |
| --- | --- | --- |
| D7 | <0.001 | 1.062 |

- **Supramammillary Nucleus (SuM)**

| **Time Point** | **p-value** | **Effect Size** |
| --- | --- | --- |
| D4 | 0.011 | 2.486 |
| D7 | <0.001 | 1.065 |
| D10 | 0.045 | 0.143 |
| D25 | 0.041 | 0.464 |

- **Dorsal Hippocampus (dHPC)**

| **Time Point** | **p-value** | **Effect Size** |
| --- | --- | --- |
| D7 | <0.001 | 0.941 |

#### **Synergistic Triplets**

- **No significant changes observed (p > 0.05).**

#### **Redundant Quadruplets**

| **Time Point** | **p-value** | **Effect Size** |
| --- | --- | --- |
| **D7** | <0.001 | 1.278 |
