# Supplementary material for: High-Order Information Analysis of Epileptogenesis in the Pilocarpine Rat Model of Temporal Lobe Epilepsy
Source: eNeuro. 2025 May 21;12(5):ENEURO.0403-24.2025. doi: 10.1523/ENEURO.0403-24.2025 (PMC12121938; doi:10.1523/ENEURO.0403-24.2025)
Supplement: Figure 12-1 — Statistics corresponding to the mutual information discussed in the sixth section of the Results entitled “Added value of higher-order interactions”. Download Figure 12-1, DOC file. [file eneuro-12-ENEURO.0403-24.2025-s013.doc]

## **Category A**

### **Sniffing Behavior**

#### **Overall Mutual Information Increase**

| **Time Point** | **p-value** | **Effect Size** |
| --- | --- | --- |
| D4 | <0.001 | 1.017 |

#### **Per Brain Region**

- **Medial Septum (MS)**

| **Time Point** | **p-value** | **Effect Size** |
| --- | --- | --- |
| D4 | <0.001 | 0.926 |

- **Dorsal Hippocampus (dHPC)**

| **Time Point** | **p-value** | **Effect Size** |
| --- | --- | --- |
| D4 | <0.001 | 0.818 |

- **Ventral Hippocampus (vHPC)**

| **Time Point** | **p-value** | **Effect Size** |
| --- | --- | --- |
| D4 | <0.001 | 0.812 |

- **Thalamus (Thal)**

| **Time Point** | **p-value** | **Effect Size** |
| --- | --- | --- |
| D4 | <0.001 | 0.704 |

#### **Pairwise Interactions**

**MS-dHPC**

| **Time Point** | **p-value** | **Effect Size** |
| --- | --- | --- |
| D4 | <0.001 | 1.254 |
| D10 | 0.002 | 0.072 (negligible) |
| D14 | <0.001 | 0.251 |

**MS-vHPC**

| **Time Point** | **p-value** | **Effect Size** |
| --- | --- | --- |
| D4 | <0.001 | 1.417 |
| D10 | <0.001 | 0.622 |

**MS-Thal**

| **Time Point** | **p-value** | **Effect Size** |
| --- | --- | --- |
| D4 | 0.008 | 1.042 |
| D10 | 0.002 | 0.468 |
| D14 | 0.036 | 0.438 |
| D25 | <0.001 | 1.060 |

**dHPC-vHPC**

| **Time Point** | **p-value** | **Effect Size** |
| --- | --- | --- |
| D4 | <0.001 | 1.013 |

**dHPC-Thal**

| **Time Point** | **p-value** | **Effect Size** |
| --- | --- | --- |
| D4 | 0.008 | 0.883 |
| D7 | <0.001 | 0.217 (small effect) |
| D10 | 0.002 | 0.283 |
| D25 | 0.041 | 0.377 |

**vHPC-Thal**

| **Time Point** | **p-value** | **Effect Size** |
| --- | --- | --- |
| D4 | <0.001 | 1.041 |
| D7 | <0.001 | 0.077 (negligible) |
| D10 | 0.025 | 0.071 (negligible) |

### **Rest Behavior**

#### **Overall Mutual Information**

| **Time Point** | **p-value** | **Effect Size** |
| --- | --- | --- |
| D10 | 0.042 | 0.066 (negligible) |
| D14 | <0.001 | 0.364 |

#### **Per Brain Region**

- **Medial Septum (MS)**

| **Time Point** | **p-value** | **Effect Size** |
| --- | --- | --- |
| D7 | 0.027 | 0.496 |
| D14 | 0.022 | 0.693 |

#### **Pairwise Interactions**

**MS-dHPC**

| **Time Point** | **p-value** | **Effect Size** |
| --- | --- | --- |
| D4 | <0.001 | 0.838 |
| D7 | <0.001 | 0.653 |
| D10 | 0.017 | 0.175 (small) |
| D14 | <0.001 | 0.762 |

**MS-Thal**

| **Time Point** | **p-value** | **Effect Size** |
| --- | --- | --- |
| D4 | 0.001 | 0.903 |
| D7 | 0.009 | 0.638 |
| D10 | 0.038 | 0.560 |

**MS-vHPC**

| **Time Point** | **p-value** | **Effect Size** |
| --- | --- | --- |
| D10 | 0.023 | 0.395 |
| D14 | 0.016 | 0.974 |

### **Sleep Behavior**

#### **Overall Mutual Information**

| **Time Point** | **p-value** | **Effect Size** |
| --- | --- | --- |
| D7 | <0.001 | 0.966 |
| D10 | <0.001 | 0.529 |

#### **Per Brain Region**

- **Medial Septum (MS)**

| **Time Point** | **p-value** | **Effect Size** |
| --- | --- | --- |
| D7 | 0.038 | 0.846 |

- **Dorsal Hippocampus (dHPC)**

| **Time Point** | **p-value** | **Effect Size** |
| --- | --- | --- |
| D10 | <0.001 | 0.747 |

- **Ventral Hippocampus (vHPC)**

| **Time Point** | **p-value** | **Effect Size** |
| --- | --- | --- |
| D10 | 0.002 | 0.416 |

## **Category B**

### **Sniffing Behavior**

#### **Overall Mutual Information Increase**

| **Time Point** | **p-value** | **Effect Size** |
| --- | --- | --- |
| D4 | <0.001 | 0.607 |

#### **Per Brain Region**

- **Medial Septum (MS)**

| **Time Point** | **p-value** | **Effect Size** |
| --- | --- | --- |
| D4 | <0.001 | 0.549 |

- **Dorsal Hippocampus (dHPC)**

| **Time Point** | **p-value** | **Effect Size** |
| --- | --- | --- |
| D4 | <0.001 | 0.552 |

- **Entorhinal Cortex (EC)**

| **Time Point** | **p-value** | **Effect Size** |
| --- | --- | --- |
| D4 | <0.001 | 0.416 |

- **Supramammillary Nucleus (SuM)**

| **Time Point** | **p-value** | **Effect Size** |
| --- | --- | --- |
| D4 | <0.001 | 0.498 |

#### **Pairwise Interactions**

**MS-dHPC**

| **Time Point** | **p-value** | **Effect Size** |
| --- | --- | --- |
| D4 | <0.001 | 0.902 |
| D7 | <0.001 | 0.282 (small) |
| D10 | <0.001 | 0.060 (negligible) |
| D14 | <0.001 | 0.001 (negligible) |
| D25 | <0.001 | 0.258 (small) |

**MS-SuM**

| **Time Point** | **p-value** | **Effect Size** |
| --- | --- | --- |
| D4 | 0.109 | 0.753 (no significant shift) |
| D10 | <0.001 | 0.617 |
| D25 | 0.007 | 0.474 |

**MS-EC**

| **Time Point** | **p-value** | **Effect Size** |
| --- | --- | --- |
| D4 | <0.001 | 0.407 |
| D7 | 0.006 | 0.201 |
| D10 | <0.001 | 0.052 (negligible) |
| D14 | <0.001 | 0.192 |
| D25 | 0.029 | 0.242 |

**SuM-dHPC**

| **Time Point** | **p-value** | **Effect Size** |
| --- | --- | --- |
| D4 | <0.001 | 0.903 |
| D7 | <0.001 | 0.272 (small) |
| D10 | <0.001 | 0.180 (small) |
| D14 | 0.006 | 0.208 (small) |
| D25 | 0.008 | 0.036 (negligible) |

**SuM-EC**

| **Time Point** | **p-value** | **Effect Size** |
| --- | --- | --- |
| D4 | <0.001 | 0.506 |
| D10 | <0.001 | 0.328 |
| D14 | <0.001 | 0.497 |

**dHPC-EC**

| **Time Point** | **p-value** | **Effect Size** |
| --- | --- | --- |
| D4 | <0.001 | 0.622 |
| D14 | 0.016 | 0.306 |

### **Rest Behavior**

#### **Overall Mutual Information**

| **Time Point** | **p-value** | **Effect Size** |
| --- | --- | --- |
| D25 | <0.001 | 0.477 |

#### **Per Brain Region**

- **Medial Septum (MS)**

| **Time Point** | **p-value** | **Effect Size** |
| --- | --- | --- |
| D7 | 0.049 | 0.419 |
| D14 | 0.041 | 0.332 |

- **Dorsal Hippocampus (dHPC)**

| **Time Point** | **p-value** | **Effect Size** |
| --- | --- | --- |
| D25 | <0.001 | 0.446 |

- **Supramammillary Nucleus (SuM)**

| **Time Point** | **p-value** | **Effect Size** |
| --- | --- | --- |
| D25 | 0.031 | 0.451 |

#### **Pairwise Interactions**

**MS-SuM**

| **Time Point** | **p-value** | **Effect Size** |
| --- | --- | --- |
| D4 | <0.001 | 0.685 |
| D10 | 0.014 | 0.468 |
| D25 | 0.031 | 0.682 |

**MS-dHPC**

| **Time Point** | **p-value** | **Effect Size** |
| --- | --- | --- |
| D4 | 0.028 | 0.168 (small) |
| D7 | <0.001 | 0.637 |
| D14 | <0.001 | 0.571 |
| D25 | 0.003 | 0.623 |

**MS-EC**

| **Time Point** | **p-value** | **Effect Size** |
| --- | --- | --- |
| D4 | 0.016 | 0.380 |
| D10 | 0.005 | 0.025 (negligible) |
| D14 | 0.025 | 0.206 (small effect) |

**SuM-dHPC**

| **Time Point** | **p-value** | **Effect Size** |
| --- | --- | --- |
| D7 | <0.001 | 0.098 (negligible) |

**dHPC-EC**

| **Time Point** | **p-value** | **Effect Size** |
| --- | --- | --- |
| D7 | 0.001 | 0.224 |

### **Sleep Behavior**

#### **Overall Mutual Information**

| **Time Point** | **p-value** | **Effect Size** |
| --- | --- | --- |
| D7 | <0.001 | 0.739 |
| D10 | 0.019 | 0.281 |

#### **Per Brain Region**

- **Medial Septum (MS)**

| **Time Point** | **p-value** | **Effect Size** |
| --- | --- | --- |
| D7 | 0.002 | 0.834 |

- **Entorhinal Cortex (EC)**

| **Time Point** | **p-value** | **Effect Size** |
| --- | --- | --- |
| D7 | 0.002 | 0.825 |

- **Dorsal Hippocampus (dHPC)**

| **Time Point** | **p-value** | **Effect Size** |
| --- | --- | --- |
| D10 | <0.001 | 0.747 |

- **Ventral Hippocampus (vHPC)**

| **Time Point** | **p-value** | **Effect Size** |
| --- | --- | --- |
| D10 | 0.002 | 0.416 |
